# Optimization of Protoplast Preparation and Establishment of Genetic Transformation System of an Arctic-Derived Fungus *Eutypella* sp.

**DOI:** 10.3389/fmicb.2022.769008

**Published:** 2022-04-06

**Authors:** Yaodong Ning, Bo Hu, Haobing Yu, Xiaoyu Liu, Binghua Jiao, Xiaoling Lu

**Affiliations:** ^1^Department of Biochemistry and Molecular Biology, College of Basic Medical Sciences, Naval Medical University, Shanghai, China; ^2^Department of Marine Biomedicine and Polar Medicine, Naval Medical Center of PLA, Naval Medical University, Shanghai, China

**Keywords:** *Eutypella* sp. D-1, protoplasts, protoplasts preparation, protoplasts regeneration, protoplasts transformation

## Abstract

Arctic-derived fungus *Eutypella* sp. D-1 has attracted wide attention due to its huge ability to synthesize secondary metabolites. However, current studies only focus on stimulating its production of new secondary metabolites by OSMAC strategies, and the relationship between secondary metabolites and biosynthetic gene clusters (BGCs) has not been explored. In this study, the preparation and regeneration conditions of *Eutypella* sp. D-1 protoplasts were explored to lay a foundation for the study of genetic transformation of this fungus. Orthogonal experiment showed that the optimal preparation conditions were 0.75 M NaCl, 20 g/L of lysing enzyme, and 20 g/L of driselase, 28°C for 6 h. The maximum yield of *Eutypella* sp. D-1 protoplasts could reach 6.15 × 10^6^ cells·ml^−1^, and the concentration of osmotic stabilizer NaCl was the most important factor for *Eutypella* sp. D-1 protoplasts. The results of FDA staining showed that the prepared protoplasts had good activity. Besides, the best protoplasts regeneration medium was YEPS, whose maximum regeneration rate is 36%. The mediums with nitrogen sources, such as SR and RM, also had good effects on the *Eutypella* sp. D-1 protoplast regeneration, indicating that nitrogen sources played an important role on the *Eutypella* sp. D-1 protoplast regeneration. Subsequent transformation experiments showed that hygromycin resistance genes (*hrg*) could be successfully transferred into the genome of *Eutypella* sp. D-1, indicating that the prepared protoplasts could meet the needs of subsequent gene manipulation and research. This study lays a foundation for the genetic transformation of *Eutypella* sp. D-1.

## Introduction

In recent years, many secondary metabolites of filamentous fungi have been found to possess unique structure and pharmacological activities, which has attracted scientists’ extensive attention (Guo et al., 2021; Ohashi et al., 2021; Qian et al., 2021). *Eutypella* species are filamentous fungi, belonging to *Ascomycetes*, *Xylariales*, and Diatrypaceae (Lu et al., 2014). They are widely distributed and found all over the world. In recent years, a variety of compounds have been isolated from *Eutypella* species, mainly including terpenoids, cytochalasins, steroids, lactones, etc. (Sun et al., 2013; Zhou et al., 2017; Zhang et al., 2020, 2021a), and it has been found that the secondary metabolites of *Eutypella* species have good antibacterial, antitumor activities (Wang et al., 2018; Zhang et al., 2021b). The diterpenoids Libertellenone H showed moderate cytotoxic activity against SW-1990, SG7901, and HeLa tumor cells (Zhang et al., 2021b). Phenochalasin B is a cytochalasins, which has strong cytotoxic activity against MCF-7, KB, Vero, HepG2, and A549 tumor cells (Liu et al., 2017). Therefore, *Eutypella* species are important sources of new drugs, showing great potential for development.

In our group, *Eutypella* sp. D-1 (D-1) was isolated from the soil of Arctic region, producing numerous structure peculiar and bioactivity diversity secondary metabolites like cytotoxicity polyhydroxyl diterpenes, cytotoxicity tyrosine-derived cytochalasins, antibacterial sesquiterpene lactone, etc (Zhang et al., 2016a, 2019; Yu et al., 2018a,b). In order to get more bioactivity metabolites, researchers mainly adopted OSMAC (one strain of many compounds) strategies, changing the culture conditions (adding precursor substances), fermentation modes (solid fermentation, liquid fermentation), etc. (Niu et al., 2018), which produce more new bioactivity metabolites like Eutypenoid D and Libertellenone H (Shen et al., 2016). However, these strategies have not been explored from the gene level of strain D-1, some silent genes may not be activated. In order to dig the biosynthetic gene clusters (BGCs) of strain D-1 better and activate its silent gene clusters to produce more bioactive secondary metabolites, the whole genome data of strain D-1 was mined. According to the bioinformatics, several biosynthetic gene clusters like PKS, terpene, NRPS-like were identified (Kumar et al., 2018), indicating strain D-1 possessing a large number of cryptic secondary metabolite biosynthesis gene clusters. How to activate these cryptic BGCs was our further studies.

Protoplasts, the cell deprived of its rigid wall but with its plasma membrane intact, offer an alternative method for genetic manipulation (Mosunova et al., 2016; Raimundo et al., 2018; Xie et al., 2020; Liang et al., 2021). This technology is a breakthrough in the genetic transformation to the fungi especially, focusing on target and modify genes to reveal the gene function more effectively (Zhang et al., 2016b; Jing et al., 2017), while, the first step is to prepare the good protoplasts. In recent years, many researchers have done a lot of research on the preparation and transformation of fungal protoplasts. The key to the preparation of fungal protoplasts is how to remove cell walls. Not only does the cell wall provide a rigid structure to the fungi, but it also protects against external factors, and it must be removed without damaging the cell membrane in order to achieve an intact protoplast. A viable protoplast can resynthesize cell wall, undergo repeated division, and regenerate the whole cell in medium. In the process of preparing fungal protoplasts, the main influencing factors are enzyme types and concentration, osmotic stabilizers, temperature, enzymatic digestion time, and fungal age (Flor-Parra et al., 2014; Wu et al., 2018; Jin et al., 2020). Different species of fungi have different conditions for protoplasts preparation (Wu and Chou, 2019). At present, there are a few reports on the preparation and regeneration studies of *Eutypella* species protoplasts to reference, which need to be explored.

In this article, we mainly studied the optimization of the protoplast preparation and regeneration of *Eutypella* sp. D-1. The PEG-mediated homologous recombination was used to test the transformation efficacy of strain D-1 protoplasts. The results showed that it could be used for the genetic transformation of strains.

## Materials and Methods

### Microorganism and Culture Conditions

The fungus *Eutypella* sp. D-1 (CCTCC NO: M 2013144) was deposited in the PDA medium at the China Center for Type Culture Collection, Wuhan, China. The mycelium of the strain was picked and inoculated in a 50-ml flask filled with PDB (24 g/L of potato dextrose broth) medium, and cultured at 28°C with 180 rpm for 3 days to obtain the seed culture.

#### Solid Medium Culture

The mycelium of the activated two- to three-generation strain was picked with a sterile bamboo skewer and inoculated into a 50-ml PDB liquid medium in a flask, and cultured at 28°C with 180 rpm for 1–7 days.

#### Liquid Medium Culture

Around 5 ml of seed culture was inoculated into 50 ml of PDB liquid medium in a flask and cultured at 28°C with 180 rpm for 1–7 days.

### Collection of Mycelium

About 10 ml of liquid medium was centrifuged at 8,000 rpm to obtain the mycelium and then washed twice with different concentrations of osmotic stabilizers.

### Preparation of Enzymatic Hydrolysate

Lysing enzyme (Lys; 0.1–0.2 g) was precisely weighed from *Trichoderma harzianum* (Sigma-Aldrich, United States) and driselase (Dri; 0.1–0.2 g) from *Basidiomycetes* sp. (Sigma-Aldrich, United States), respectively (Table 1). Around 10 ml of different concentrations (0.7–0.8 M) of osmotic stabilizers (NaCl, KCl, MgSO_4_, sucrose, sorbitol, and mannitol) was added, vortexed, and mixed until dissolved, then filtered through a 0.22-μm membrane filter and prepared for use.

**Table 1:** Enzyme combinations for strain D-1 protoplast preparation.

S. no	Enzyme combinations
1	20 mg/ml of lysing enzyme + 20 mg/ml of driselase
2	10 mg/ml of lysing enzyme + 10 mg/ml of driselase
3	10 mg/ml of lysing enzyme + 20 mg/ml of driselase
4	15 mg/ml of lysing enzyme + 15 mg/ml of driselase
5	20 mg/ml of lysing enzyme + 10 mg/ml of driselase

### Preparation and Counting of Protoplasts

The prepared different enzymatic hydrolysate was added to the mycelium and incubated in a flask at 120 rpm for 4–6 h. The reaction solution was filtered through four layers of wiped paper, and the filtrate was centrifuged at 2,500 rpm for 10 min. The precipitate was the protoplasts, which were washed with different concentrations of osmotic stabilizers and then centrifuged at 2,500 rpm for 10 min, repeated twice. The protoplasts were resuspended with appropriate amount of osmotic stabilizers. The protoplasts were counted using a hemocytometer plate in bright field under a ×400 microscope (Olympus IX71, Japan).

### Fluorescein Diacetate Staining Method to Verify Protoplasts Activity

The non-fluorescent fluorescein diacetate (FDA) is broken down by esterases upon entering into the protoplasts to produce fluorescent polar substances. The active protoplasts can produce yellow-green fluorescence under fluorescence microscopy, while inactive protoplasts cannot break down FDA and do not produce fluorescence.

Fluorescein diacetate (5 mg) was dissolved in 1 ml of acetone and stored at 4°C in a refrigerator. The FDA solution was added to the protoplasts solution, and the final concentration was maintained at 100 μg/ml. Protoplasts were observed using a ×400 fluorescence microscope under 490-nm blue light.

### Regeneration of Protoplasts

The regeneration medium composition is listed in Table 2. The regeneration medium was sterilized at 115°C for 30 min and then cooled to 55°C. The protoplast suspension was slowly added to the regeneration medium and mixed, poured into the cell culture dish, and incubated in a constant temperature incubator at 28°C to observe the regeneration. The protoplast regeneration rate is calculated as follows: (A − B)/C * 100% (A is the number of colonies in the experimental group, B is the number of colonies in the control group, and C is the number of protoplasts).

**Table 2:** Components of strain D-1 regeneration medium.

Regeneration medium	Components	Reference
SR	Yeast extract 0.1%, casein hydrolysate 0.1%, sucrose 1 M, and agar 0.7%	This study
YEPS	Yeast extract 3.5 g/L, tryptone 5 g/L, sucrose 200 g/L, and agar 15 g/L	This study
SH	Sucrose 200 g/L, Hepes 2.5 g/L, CaCl_2_ 0.1 g/L, and agar 15 g/L	This study
PDA	Potato dextrose broth 24 g/L, agar 15 g/L	This study
RM	Maltose 1%, glucose 0.4%, yeast extract 0.4%, mannitol 0.6 M, and agar 0.8%	Jin et al., 2020

### Transformation of Strain D-1 Protoplasts

*Eutypella* sp. D-1 protoplasts and hygromycin resistance gene (*hrg*) fragments (Supplementary Figures S1, S2; Supplementary Table S1) were mixed, placed on ice for 15 min, and 800 μl of PEG4000 solution was added to the mixture, standing at room temperature for 10 min. The mixture was slowly added to 50 ml of 50°C YEPS medium, gently mixed well, poured into a culture dish, and cultured at 28°C until protoplast recovery. Hygromycin B (50 μl) was added to 50 ml of PDA medium, the upper plate was inverted, and it was incubated at 28°C. The growing mycelium was picked and transferred three times in PDA medium containing hygromycin B. Mycelial DNA was extracted, and PCR was performed using hygromycin resistance gene primers with the following conditions: 94°C for 10 min, 35 cycles of 94°C for 30 s, 50°C for 30 s, 72°C for 2 min, and 72°C for 7 min. PCR products were subjected to agarose gel electrophoresis, and bands of interest were visualized under UV light.

## Results

### Effect of Fungal Age and Osmotic Stabilizer on Strain D-1 Protoplast Preparation

According to the previous references (Flor-Parra et al., 2014; Wu et al., 2018; Wu and Chou, 2019; Jin et al., 2020), the fungal age and mycelium status were very important in the preparation of fungal protoplasts, which is the basis to prepare the protoplasts successfully. If the culture time of mycelium was too short, mycelium does not proliferate, leading to too little mycelium and insufficient protoplasts. While the culture time of mycelium was too long, the mycelium aged, and the cell wall was thick, which is difficult to form a single protoplast by enzymolysis. Fresh and tender mycelium is a good raw material for the preparation of protoplasts.

Due to the lack of the cell wall protection, the protoplasts need to maintain cell morphology in a certain concentration of osmotic stabilizer. Protoplasts are very sensitive to different types and concentrations of osmotic stabilizers. If the osmotic pressure is too high, it can make protoplasts shrink. On the contrary, the protoplasts would burst, which will both degrade the quality of the protoplast. The best kind and concentration of osmotic stabilizer is the priority to the protoplast system. We first investigated the fungal age and osmotic pressure stabilizer.

#### Effect of Fungal Age and Mycelium Status on Strain D-1 Protoplast Preparation

**Figure 1:**
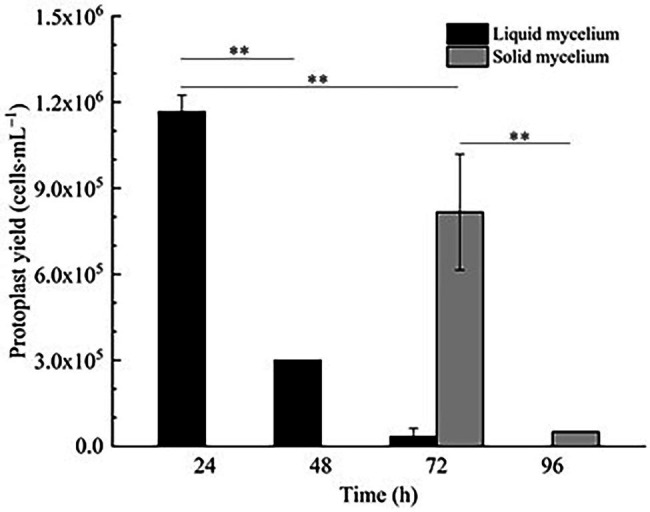
Effect of fungal age and mycelium status on strain D-1 protoplast preparation (^**^p < 0.01).

**Figure 2:**
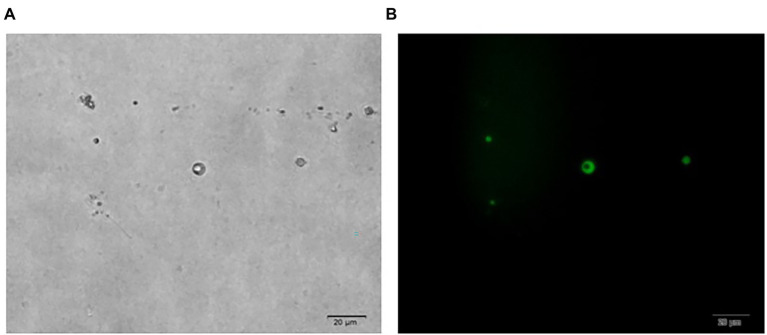
Microscopic view of strain D-1 protoplasts (×400). **(A)** Bright field. **(B)** Fluorescence field.

In Figure 1, it is shown that when liquid mycelium is inoculated, the maximum number of protoplasts can be obtained after 24 h, which was 1.17 × 10^6^ cells·ml^−1^. The number of protoplasts decreased sharply with the time going on, and almost no protoplasts were produced after 72 h. After 72 h, the cell wall of the mycelium gradually thickened. Although the number of mycelium increased, the thickening of the cell wall made it difficult to be enzymatically hydrolyzed, resulting in a significant decrease in the number of protoplasts produced. When solid mycelium was inoculated and cultured in the liquid medium, it grew slowly before 72 h, with too few mycelium for enzymatic hydrolysis, but at the 72nd hour, mycelium grew rapidly. At this time, the largest number of protoplasts was obtained at about 8.17 × 10^5^ cells·ml^−1^. After 72 h, the mycelium further grew rapidly, but the number of protoplasts decreased sharply. Maybe the long culture time led to the thickening of the cell wall of mycelium; it is difficult to enzymatically hydrolyze. However, in reality, the number of protoplasts from the liquid mycelium culture was more than that from the solid mycelium culture; the density of solid mycelium was much less than that of liquid mycelium; using solid mycelium inoculation cultivation is better and easier for digestion. Therefore, collecting a large number of mycelium in the liquid mycelium culture was chosen to satisfy the amount of protoplast preparation. Meanwhile, it can save time and speed up the experimental process. After the enzymatic hydrolysis of strain D-1 mycelium, a large number of single-celled protoplast can be obtained, whose activity was very important to the subsequent genetic and transformation experiments. Next, the activity of the prepared protoplasts was assayed. As shown in the Figure 2, After FDA staining, the protoplasts showed full and transparent spheroids with different sizes under bright-field observation (Figure 2A), while the prepared protoplasts emitted strong yellow-green fluorescence under fluorescence microscope (Figure 2B), which show that it had good activity and could be used for subsequent transformation and other studies.

#### Effect of Osmotic Stabilizer on Strain D-1 Protoplast Preparation

**Figure 3:**
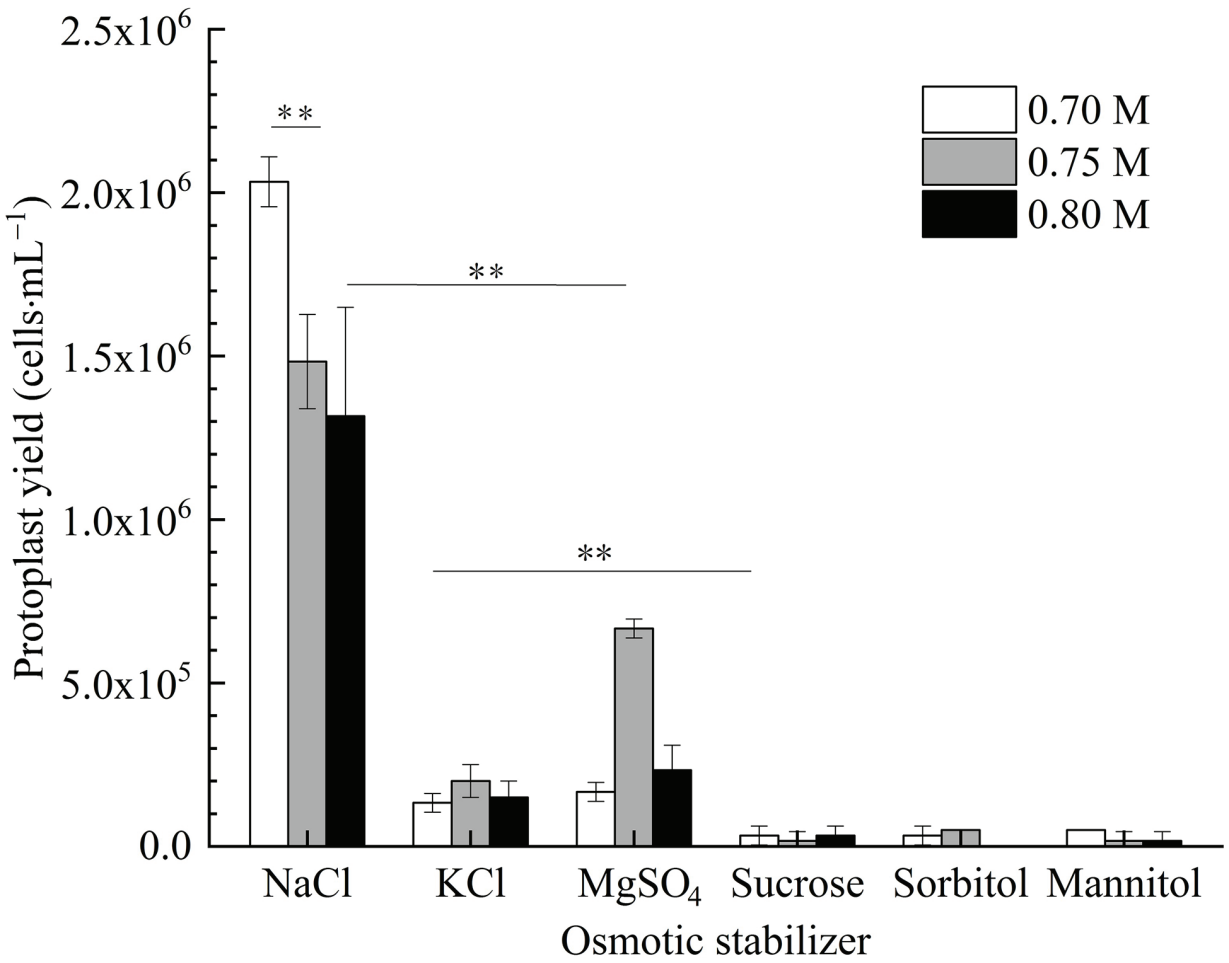
Production of strain D-1protoplasts in different osmotic stabilizers (^**^p < 0.01).

In Figure 3, it is shown that the number of protoplasts of strain D-1 in inorganic salt (NaCl, KCl, and MgSO_4_) osmotic stabilizers was significantly more than that in organic solution (sucrose, sorbitol, and mannitol) osmotic stabilizers. There were nearly no protoplasts under the organic solution as osmotic stabilizers, which indicated that strain D-1 protoplasts are more likely to maintain good cell morphology in the presence of inorganic salts. In the presence of inorganic salt osmotic pressure stabilizer, the number of protoplasts obtained in NaCl osmotic stabilizer is more than that in KCl and MgSO_4_ osmotic stabilizer at the same concentration. The highest protoplasts yield in 0.7 M NaCl osmotic stabilizer is 2.03 × 10^6^ cells·ml^−1^, which was significantly higher than that of 0.75 and 0.8 M NaCl. In the following study, NaCl solution was selected as the osmotic stabilizer to investigate various conditions.

### Single Factor Test of Strain D-1 Protoplast Preparation

Besides fungal age and mycelium status, some other factors like concentration of osmotic stabilizer, enzyme combination and the concentrations, enzymatic hydrolysis time, and temperature will also play a great impact on the yield of protoplasts.

#### Effect of NaCl Concentration on Strain D-1 Protoplast Preparation

**Figure 4:**
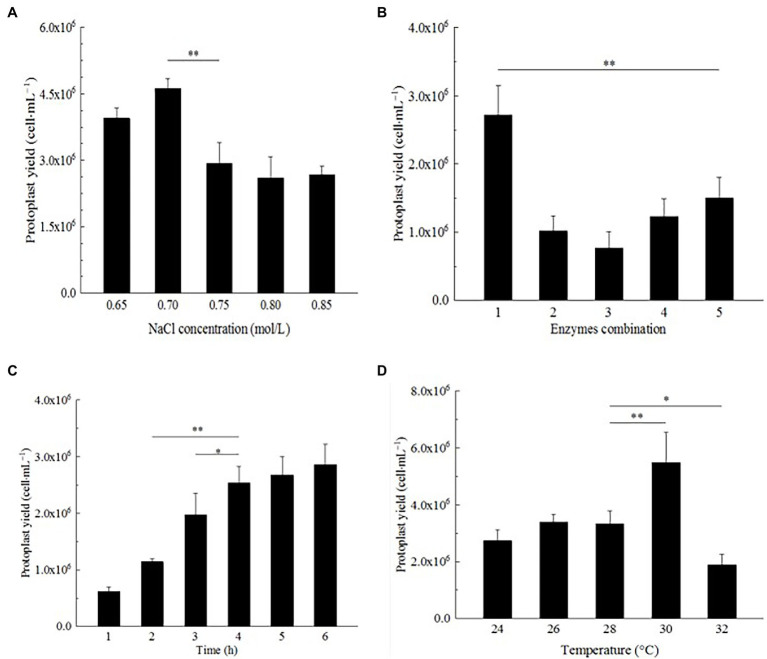
Optimization of preparation conditions of strain D-1 protoplasts. **(A)** NaCl concentration. **(B)** Enzyme combination. **(C)** Enzymatic hydrolysis time. **(D)** Enzymatic hydrolysis temperature (^**^p < 0.01; ^*^p < 0.05).

In the *Effect of osmotic stabilizer on strain D-1 protoplast preparation* section, NaCl was an excellent osmotic stabilizer to prepare strain D-1 protoplasts. The influence of different concentrations of NaCl on the preparation of strain D-1 protoplasts was further investigated. In Figure 4A, strain D-1 protoplasts have different sensitivities to different concentrations of NaCl. The highest protoplasts yield was 4.62 × 10^6^ cells·ml^−1^ at a concentration of 0.7 M NaCl, while the yield decreased at 0.65 M NaCl. With the NaCl concentration increasing, the protoplast yield decreased. NaCl (0.7 M) is the optimal osmotic stabilizer for the preparation of protoplasts.

#### Effect of Enzyme Combination on Strain D-1 Protoplast Preparation

Different fungi have different selectivity and sensitivity to the cell wall lyase because of different cell wall composition. According to the references (Flor-Parra et al., 2014; Jin et al., 2020), most traditional enzymes are lysing enzyme, driselase, lyticase, yatalase, etc., and the combination of multiple enzymes can achieve a better effect. In this study, lysing enzyme and driselase were used in combination (Table 1). In Figure 4B, it can be seen that the number of protoplasts lysing by different combinations of the enzymes is significantly different. Mycelium were lysed with a combination of enzyme (20 mg/ml of lysing enzyme + 20 mg/ml of driselase), and the protoplasts were as high as 2.72 × 10^6^ cells·ml^−1^, which was significantly larger than the number of protoplasts in other treatments, in which there was a positive correlation between protoplast yield and enzyme concentration. There were significant differences in the yield of protoplasts among different enzyme combinations, suggesting that strain D-1 had a certain selectivity to the different enzymes because of its composition of the cell wall. Different enzymes may act on the different sites and sequences of the cell wall, resulting in great differences in the number of protoplasts hydrolyzed by enzymes.

#### Effect of Enzymatic Hydrolysis Time on Strain D-1 Protoplast Preparation

The enzymatic hydrolysis time also had a great influence on the formation of strain D-1 protoplasts (Figure 4C). It is shown that with the increase in enzymatic hydrolysis time, from 1 to 4 h, the strain D-1 protoplasts had a rapid growth rate. At the fourth hour, the yield of protoplasts could reach 2.54 × 10^6^ cells·ml^−1^. With the further extension of enzymatic hydrolysis time, the growth rate of protoplasts significantly slowed down, and there was no significant difference in the yield of protoplasts from 4 to 6 h, indicating that the protoplasts had basically been released at the fourth hour. Besides, it is shown that the protoplast yield from 4 to 6 h neither increased nor decreased, which indicated that NaCl osmotic stabilizer provided a stable environment to keep the osmotic pressure of protoplasts stable and maintain the good state of cells for a long time.

**Figure 5:**
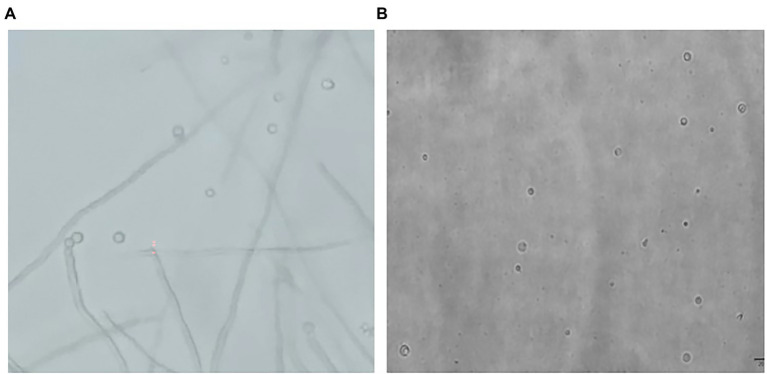
The process of protoplasts releasing **(A)** and morphology **(B)** of strain D-1.

At the same time, the process of strain D-1 protoplast generation (Figure 5A) was observed. The top of the mycelium expanded, and the mycelial wall at the cleavage site was broken under the actions of enzymes. The protoplast cells fell off from the cleavage site one by one into the osmotic stabilizer. Due to the lack of cell wall protection, protoplasts immediately expanded into round spheres after releasing with different sizes (Figure 5B). The density of mycelium decreased, with only fragments of mycelium remaining after enzymatic hydrolysis.

#### Effect of Enzymatic Hydrolysis Temperature on Strain D-1 Protoplast Preparation

Each enzyme has its most suitable enzymatic reaction temperature in the reaction. The enzymatic hydrolysis temperature is a relatively important factor for the preparation of strain D-1 protoplasts. In Figure 4D, it could be seen that the yield of protoplasts does not change obviously with the increasing enzymatic hydrolysis temperature at 24–28°C, which indicated that the temperature in this range is not the best enzymatic reaction temperature. Compared with other groups, the yield of protoplasts increased significantly at 30°C, reaching 5.48 × 10^6^ cells·ml^−1^. However, with the further increase in the enzymatic hydrolysis temperature, the yield of protoplasts decreased significantly, indicating that the enzyme system was more sensitive to temperature. In the preparation of strain D-1 protoplast, 30°C was the most suitable enzymatic hydrolysis temperature.

### Orthogonal Test of Strain D-1 Protoplast Preparation

According to the results of the single-factor experiment, NaCl concentration, enzyme combination, enzymatic hydrolysis temperature, and time are all the important factors of the preparation of strain D-1 protoplasts. These factors are often complex synergistic or inhibited, so it is necessary to use orthogonal experiment to investigate the optimal preparation conditions of protoplasts (Table 3). According to the orthogonal experiment results (Table 4), the NaCl concentration (factor A), enzyme combination (factor B), enzymatic hydrolysis time (factor C), and enzymatic hydrolysis temperature (factor D) are the order of primary and secondary factors. The optimal reaction condition was as follows: NaCl concentration of 0.75 M, enzyme combination of 20 mg/ml of lysing enzyme and 20 mg/ml of driselase, enzymolysis time of 6 h, and enzymolysis temperature of 28°C (A3B1C3D2). The optimal reaction condition was just No. 7 of the experimental condition with the maximum protoplasts yield of 6.15 × 10^6^ cells·ml^−1^.

**Table 3:** Orthogonal experimental design.

S. no	A	B	C	D
	NaCl (M)	Enzymes	Time (h)	Temperature (°C)
1	0.65	20 mg/ml of Lys + 20 mg/ml of Dri	4	26
2	0.7	15 mg/ml of Lys + 15 mg/ml of Dri	5	28
3	0.75	20 mg/ml of Lys + 10 mg/ml of Dri	6	30

**Table 4:** The orthogonal experiment results of strain D-1 protoplast preparation.

S. no	A	B	C	D	
	NaCl (M)	Enzymes	Time (h)	Temperature (°C)	Protoplast yield (10^6^ cells·ml^−1^)
1	0.65	20 mg/ml of Lys + 20 mg/ml of Dri	4	26	1.575 ± 0.197
2	0.65	15 mg/ml of Lys + 15 mg/ml of Dri	5	28	1.242 ± 0.208
3	0.65	20 mg/ml of Lys + 10 mg/ml of Dri	6	30	1.292 ± 0.139
4	0.7	20 mg/ml of Lys + 20 mg/ml of Dri	5	30	2.067 ± 0.331
5	0.7	15 mg/ml of Lys + 15 mg/ml of Dri	6	26	2.000 ± 0.354
6	0.7	20 mg/ml of Lys + 10 mg/ml of Dri	4	28	1.517 ± 0.280
7	0.75	20 mg/ml of Lys + 20 mg/ml of Dri	6	28	6.150 ± 1.427
8	0.75	15 mg/ml of Lys + 15 mg/ml of Dri	4	30	2.000 ± 0.235
9	0.75	20 mg/ml of Lys + 10 mg/ml of Dri	5	26	1.792 ± 0.196
K1	4.109	9.792	5.092	5.367	
K2	5.584	5.242	5.101	8.909	
K3	9.942	4.601	9.442	5.359	
R	5.833	5.191	4.350	3.550	

### Effect of Regeneration Medium on Strain D-1 Protoplast Regeneration

**Figure 6:**
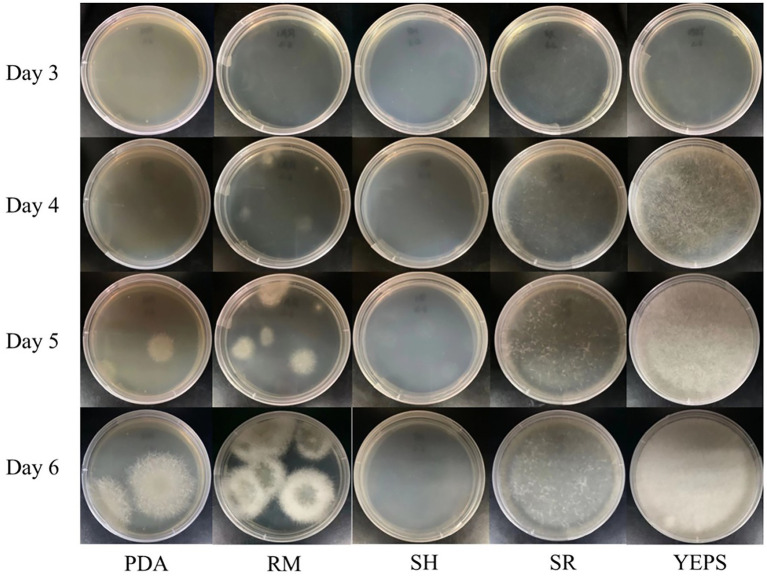
Effect of strain D-1 protoplast regeneration in different regeneration media.

**Figure 7:**
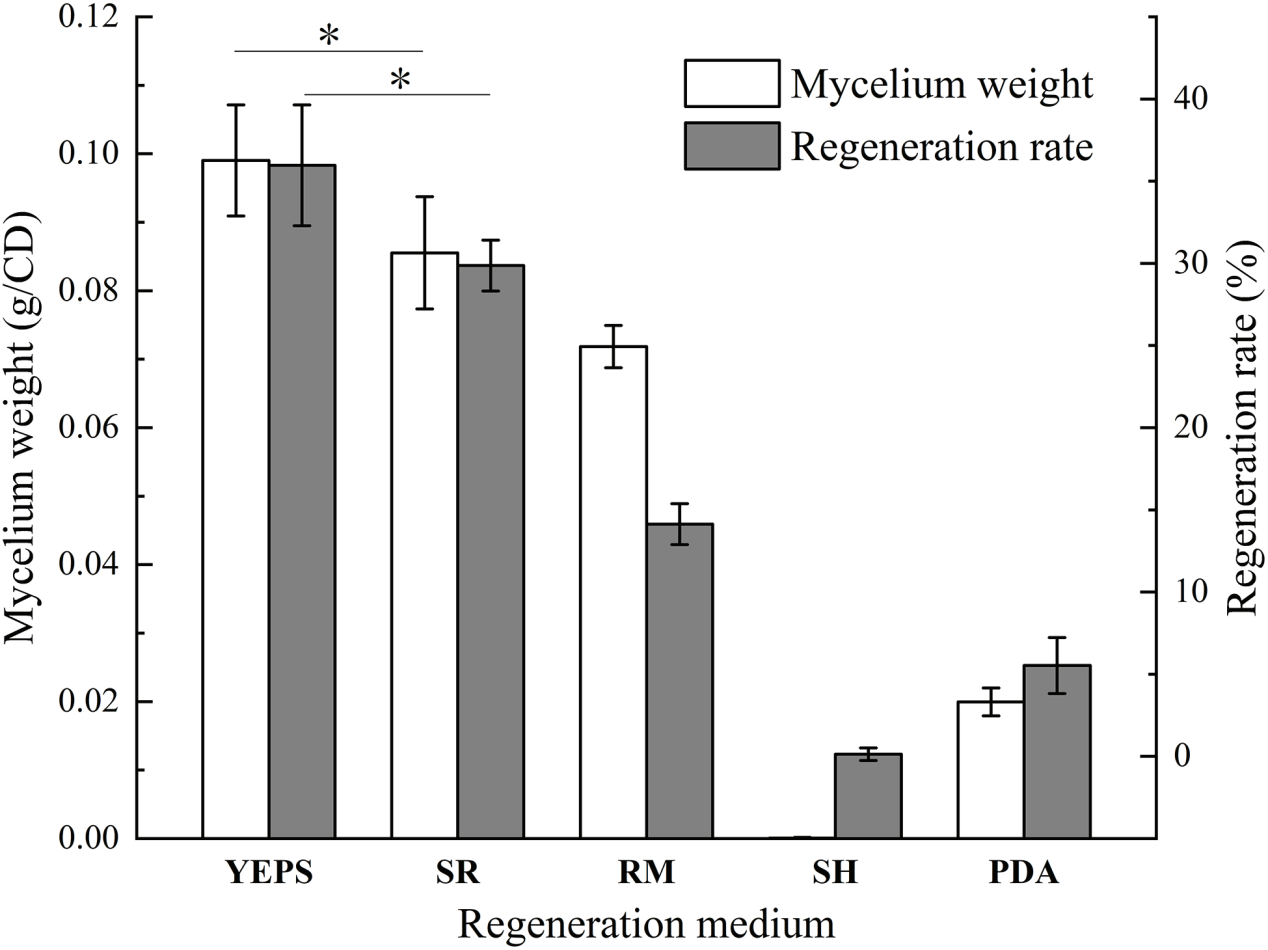
The wet weight of regeneration mycelium and protoplast regeneration rate of strain D-1 (^*^p < 0.05).

Due to the lack of cell wall, protoplasts are more fragile than mycelium and very vulnerable to the external environment, affecting the activity. How to regenerate and proliferate protoplasts quickly and efficiently is the key point to determine whether the protoplasts can be utilized. In this study, the five regeneration media, which had been reported to be good for fungal protoplast regeneration were chosen (Table 2). The resuscitation effect was observed every day to judge which regeneration medium is more suitable for strain D-1. As shown in Figure 6, almost no mycelium appeared in the five media in the first 3 days; only the tiny young mycelium could be found inside the SR and YEPS medium, indicating that the mycelium slowly adapted to the culture environment in the first 3 days. On the fourth day, mycelia were recovered in all the five media with varying degrees, but the mycelia were rapidly germinated in YEPS medium, a small number of mycelia were germinated in SR medium, and almost no mycelia were germinated in the other three media. From the fifth to the sixth day, the mycelia all underwent rapid germination in all the media except in the SH medium, among which the mycelia in YEPS medium had the fastest germination rate and could almost cover the whole Petri dish on the fifth day. In PDA and RM medium, the mycelia grew in the form of colonies, and the mycelia grew unevenly, obviously showing that the protoplasts only partially regenerated. However, almost no mycelia germinated in the SH medium. After weighing the mycelia cultured for 10 days, it was found that the largest number of mycelia was regenerated in the YEPS medium of 0.09 g/culture dish (Figure 7). In addition, more mycelia were regenerated in the SR and RM media, while no mycelium was observed in the SH medium. In terms of protoplast regeneration rate, protoplasts in the YEPS regeneration medium got the highest regeneration rate of 36%, followed by SR and RM, which also have relatively good regeneration rates of 30 and 14%, respectively (Figure 7). There was no regenerated mycelium observed in the SH medium, wherein the regeneration rate was very low. In general, YEPS is the best regeneration medium for strain D-1 in terms of mycelium regeneration quantity and protoplast regeneration rate. The regeneration medium with good protoplast regeneration cannot only improve the success of subsequent transformation experiments but also shorten the growth time of the fungus and speed up the experimental process.

### Evaluation of the Transformation Efficacy of Strain D-1 Protoplast

**Figure 8:**
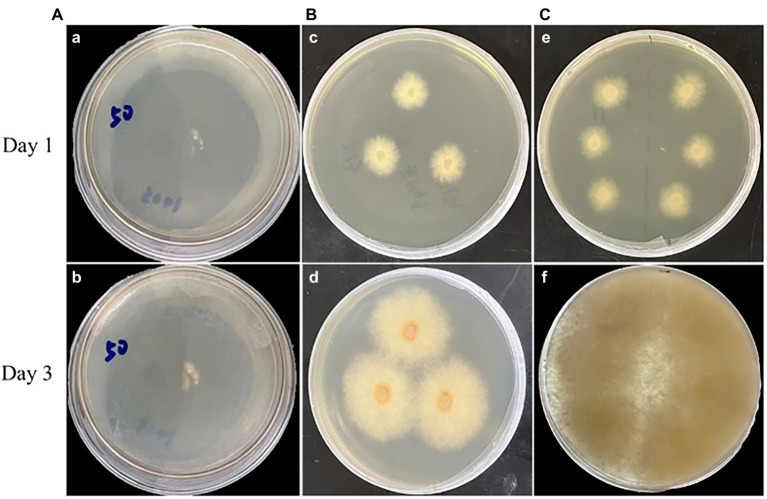
Strain D-1 was grown on PDA medium. **(A)** Wild-type (WT) strain in medium containing hygromycin B (HmB). **(B)** Transformant in medium not containing HmB. **(C)** Transformant in medium containing HmB.

**Figure 9:**
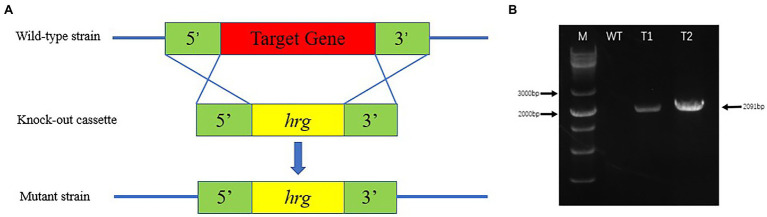
Identification of strain D-1 transformants. **(A)** Transformation strategy. **(B)** Identification of transformants (M, marker; WT, wild-type strain; T1, transformant 1; and T2, transformant 2).

Highly efficient genetic transformation depended on the high-quality protoplasts. In this study, hygromycin resistance gene was used to transfer into strain D-1 genome to evaluate the quality of the strain D-1 protoplasts. Hygromycin B (HmB) is an antibiotic with broad-spectrum activity against prokaryotes and eukaryotes; D-1 cannot grow in the medium supplemented with HmB. If the hygromycin resistance gene is successfully transferred into the strain D-1 genome, strain D-1 can grow normally in the medium with HmB. As shown in Figure 8, the wild-type (WT) D-1 strain is difficult to grow in the medium supplemented with HmB (Figure 8A), while the successfully transformed D-1 strain can grow well in the medium with HmB (Figure 8C), and the growth rate is basically the same as that originally in the PDA medium without HmB (Figure 8B). In the further verification experiment, the hygromycin resistance gene fragment was successfully amplified in the genome of strain D-1 through PCR (Figure 9; Supplementary Table S3). The above experiments fully proved that the strain D-1 protoplasts prepared have high quality and can successfully realize genetic transformation, which provides a means for subsequent gene research.

## Discussion

To further investigate the relationship between *Eutypella* sp. D-1 secondary metabolites and their biosynthetic gene clusters, a strain D-1 genetic and translational manipulation system was required. The method of PEG-mediated protoplast transformation of fungi is currently a more mature and effective way of performing epigenetic manipulation. Therefore, it is important to establish the method of preparation and regeneration of *Eutypella* sp. D-1 protoplasts as a basis for studying the direction of its biosynthesis.

According to the orthogonal result, there are two key steps in protoplast preparation of strain D-1: how to lyse the cell wall by enzymes and maintain the stability of osmotic pressure. The cell wall composition of strain D-1 may be complex, and the protoplast yield can be increased by adjusting the content of lysing enzyme and driselase. Different enzymes act on different substrates, and the interaction between enzymes may also affect the enzymolysis effect. Both enzymes used in this study were complex enzyme systems, in which the lysing enzyme had better enzymolysis effect on the cell walls containing cellulose, chitin, and other components, while the driselase had better enzymolysis effect on the cell walls containing laminarin, xylan, and cellulose. Compared with the single-enzyme system, the multi-enzyme system can enzymolyse the complex cell wall more efficiently. The released protoplasts need to keep their morphology and function normal in a stable osmotic environment due to the lack of protective effect of the cell wall. Strain D-1 protoplasts could only live in a certain concentration range of NaCl to maintain its stable state. Besides, compared with the enzyme combinations and osmotic stabilizer, the influence of enzymatic hydrolysis time and temperature is relatively small, but there are some difficulties in the experiment. The filamentous fungi *Eutypella* sp. D-1 cannot distribute evenly in liquid medium, which is not accurate in evaluating the mycelium concentration by optical density (OD) value, and it is difficult to establish the standard curve to illustrate the relationship between the number of mycelium and the protoplasts. We can only unify the experimental condition of 10% mycelial inoculum in 50 ml of liquid medium and 20-h culture to collect all the mycelium for the preparation of protoplasts. According to our experience, the protoplast yield was maintained at about 6 × 10^6^ cells·ml^−1^ under this condition, which can be used as the protoplast forming rate standard for the subsequent transformation experiment.

The best medium for regeneration of strain D-1 protoplasts was YEPS. Compared with the protoplast regeneration effect and different medium compositions, first, it is shown that all the media contained a large number of different carbon sources to meet the nutrient requirements for protoplast regeneration. However, although the SH medium contained a large number of carbon sources, the protoplasts did not have a good regeneration effect, which means that carbon sources was not the key point to regenerate the protoplasts. Second, the regeneration medium YEPS, SR, and RM with good recovery effect have different contents of nitrogen source, such as yeast extract and tryptone. The nitrogen-containing medium, such as SR and RM, also had better effect on the regeneration of strain D-1 protoplasts, indicating that the nitrogen source was the key point in the regeneration of strain D-1 protoplasts. The main function of the nitrogen source is to synthesize protein, which plays a very important role in the process of microbial proliferation. Maybe the nitrogen source can promote cell division during strain D-1 protoplast regeneration, making mycelium proliferate rapidly. However, the most regenerated colonies of some fungi, such as yeast and *Penicillium*, grew in a single colony state, and there are obvious boundaries between the colonies, which could be facilitated using the formula to calculate the regeneration rate of the protoplasts. However, when our filamentous fungi D-1 regenerated in the regeneration medium, it was still in the state of mycelium without growing and forming a single colony, especially in the YEPS and SR media. In the early stage of protoplast regeneration, mycelium, which looks like feather, does appear in the culture medium. These mycelium, without forming round colonies, exist relatively independently, which could be regarded as a single colony. Then we calculated the protoplast regeneration rate according to the number of these single colonies. In this article, we used this method to evaluate the protoplast regeneration rate of the different kinds of the regeneration media. The results showed that the optimal regeneration rate was 36% of YEPS medium. In general, YEPS is the best regeneration medium for strain D-1 in terms of mycelial regeneration quantity and protoplast regeneration rate. The YEPS medium will be used as the regeneration medium of protoplasts in subsequent experiments.

Subsequent transformation experiments showed that hygromycin resistance genes could be successfully transferred into the genome of strain D-1. However, since the growth process of our filamentous fungi may cross together, a clear boundary cannot be formed between colonies, which brings some difficulties to the calculation of transformation efficiency. However, we still estimate that about 20 transformants can be obtained from the 2 μg of resistance gene fragment, and 80% of the transformants are positive after PCR identification. Because we will use this method to knock out the filamentous fungi gene later, it can meet the requirements of our subsequent experiments. In short, the prepared protoplasts could meet the needs of subsequent gene manipulation and research.

## Conclusion

In this paper, we investigated the protoplast preparation and regeneration methods of the Arctic-derived fungus *Eutypella* sp. D-1. The optimal enzymatic time for the preparation of strain D-1 protoplast mycelium was specified as no more than 24 h in liquid medium. The single-factor experiment clarified the factors that had a strong influence on the preparation of strain D-1 protoplasts, including the types and concentration of osmotic stabilizer, enzyme combination, enzymatic digestion time and temperature, and other elements. The orthogonal experiment showed the optimal preparation conditions of 0.75 M NaCl, 20 g/L of lysing enzyme, and 20 g/L of driselase, enzymatic hydrolysis temperature of 28°C, and enzymatic hydrolysis for 6 h. The maximum preparation amount of the strain D-1 protoplasts could reach 6.15 × 10^6^ cells·ml^−1^. The most important factor for the preparation of strain D-1 protoplasts was the concentration of the osmotic stabilizer NaCl. The FDA staining method showed that the prepared protoplasts had good activity. Transformation experiments showed that hygromycin resistance genes could be successfully transferred into the genome of strain D-1. In summary, this study investigated the preparation and regeneration conditions of strain D-1 protoplasts, which laid the foundation for studying the genetic transformation of *Eutypella* sp. Besides, it also provides a reference for the preparation and transformation of the protoplasts of the *Eutypella* species.

## Data Availability Statement

The original contributions presented in the study are included in the article/Supplementary Material, further inquiries can be directed to the corresponding authors.

## Author Contributions

YN: conceptualization, methodology, software, formal analysis and investigation, writing—original draft preparation, and writing—review and editing. BH: software and investigation. HY: resources, formal analysis, and investigation. XLi: resources and investigation. BJ: conceptualization, methodology, and writing—review and editing. XLu: conceptualization, methodology, writing—original draft preparation, and writing—review and editing. All authors contributed to the article and approved the submitted version.

## Funding

This research was funded by National Key R&D Program of China (2019YFC0312504 and 2018YFC1406702).

## Conflict of Interest

The authors declare that the research was conducted in the absence of any commercial or financial relationships that could be construed as a potential conflict of interest.

## Publisher’s Note

All claims expressed in this article are solely those of the authors and do not necessarily represent those of their affiliated organizations, or those of the publisher, the editors and the reviewers. Any product that may be evaluated in this article, or claim that may be made by its manufacturer, is not guaranteed or endorsed by the publisher.
