# Supplementary figures and images for: Optimization of Protoplast Preparation and Establishment of Genetic Transformation System of an Arctic-Derived Fungus Eutypella sp
Source: Front Microbiol. 2022 Apr 6;13:769008. doi: 10.3389/fmicb.2022.769008 (PMC9019751; doi:10.3389/fmicb.2022.769008)

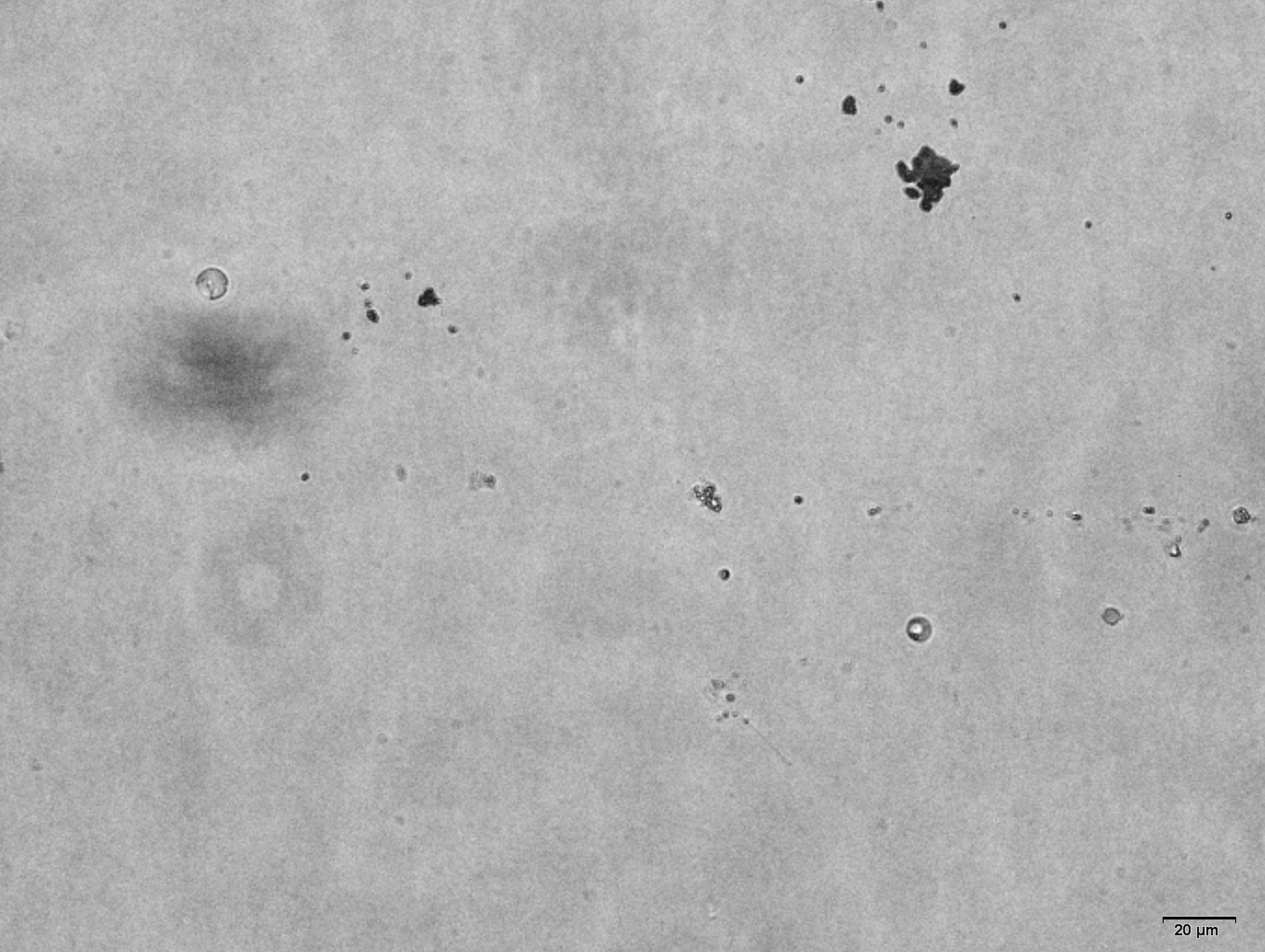

Supplement: Supplementary file 1 [file Data_Sheet_1.ZIP › Primary Data/Primary Data/Figure2/a.tif]

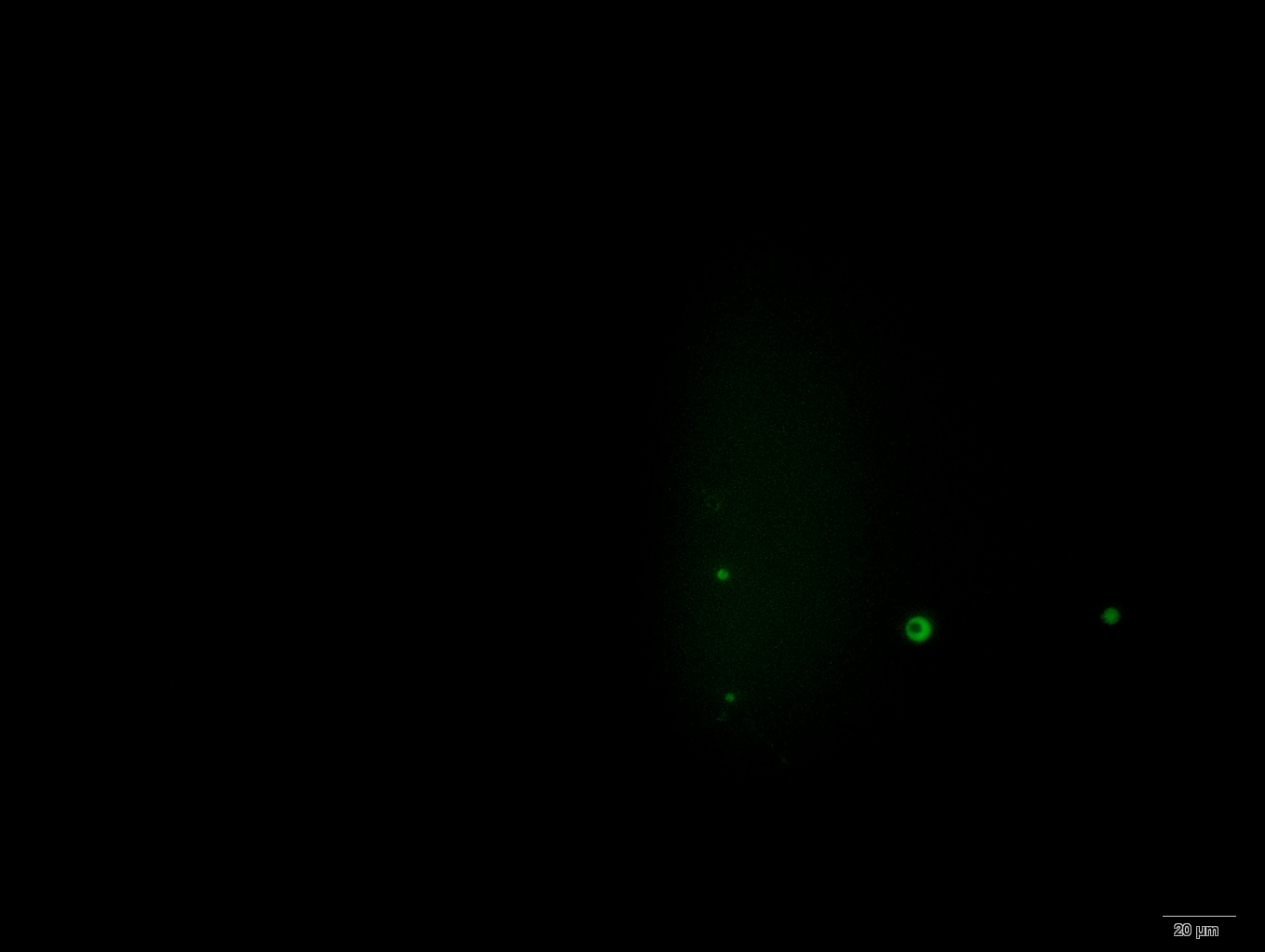

Supplement: Supplementary file 1 [file Data_Sheet_1.ZIP › Primary Data/Primary Data/Figure2/b.tif]

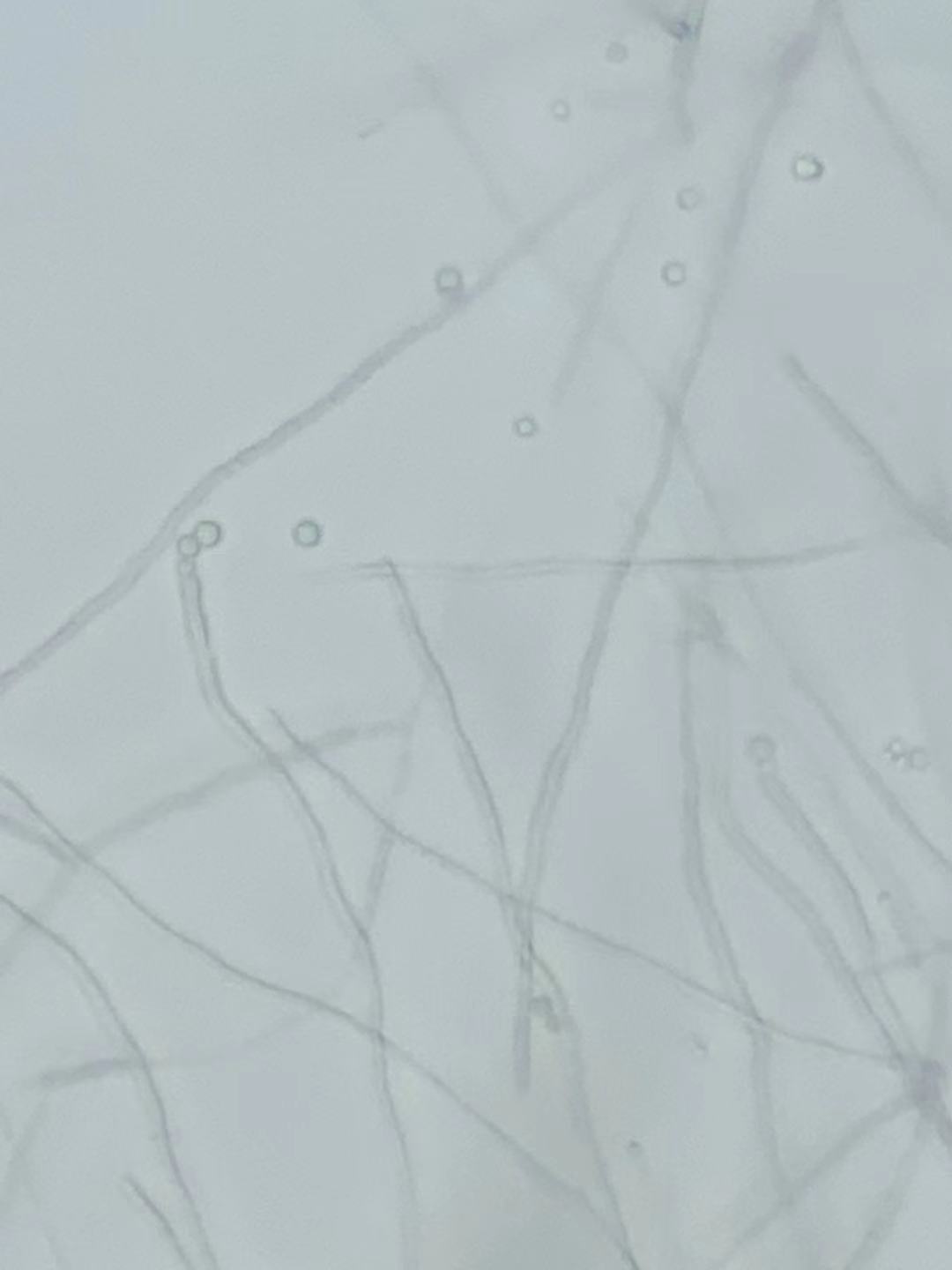

Supplement: Supplementary file 1 [file Data_Sheet_1.ZIP › Primary Data/Primary Data/Figure5/a.jpg]

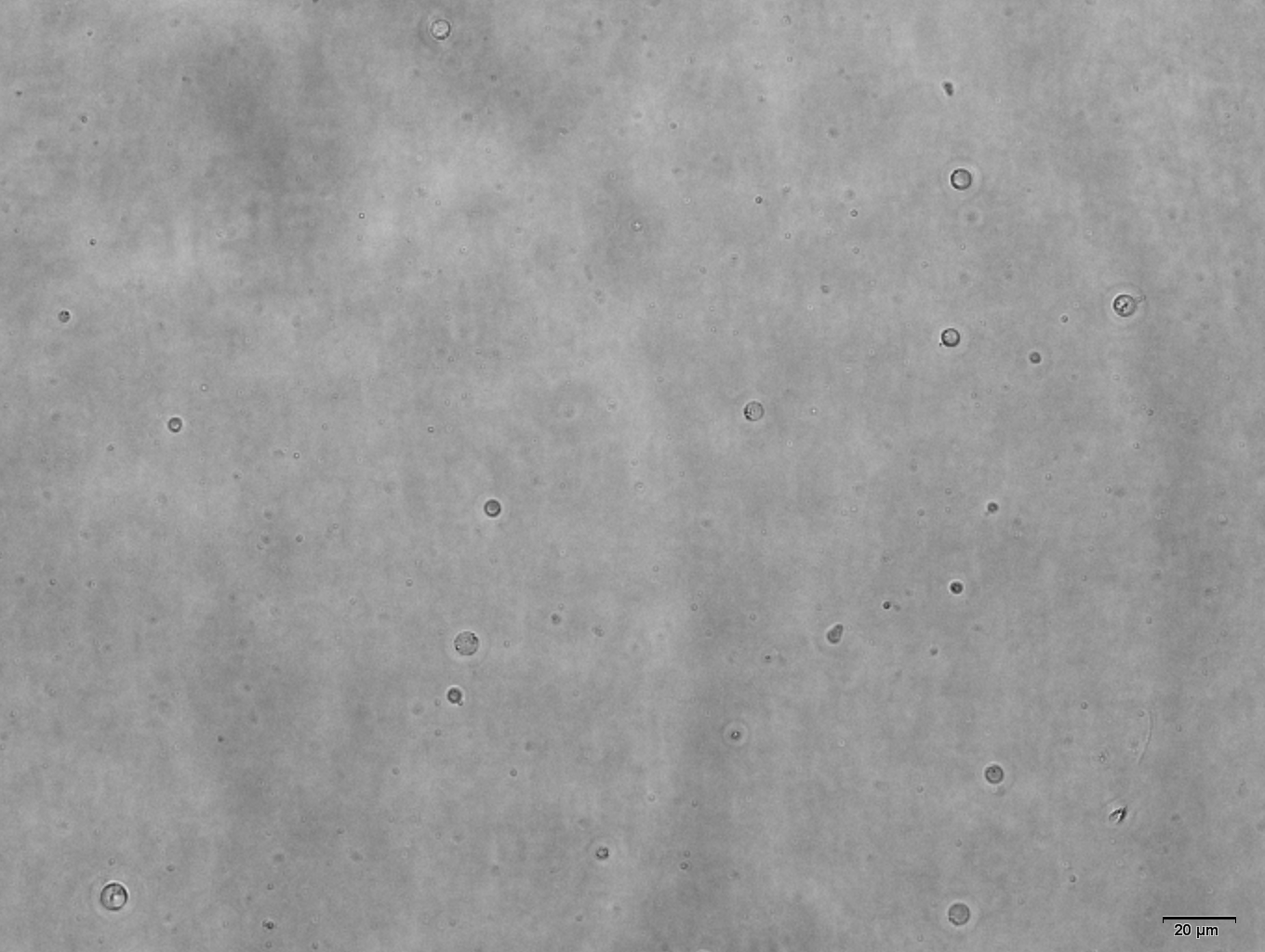

Supplement: Supplementary file 1 [file Data_Sheet_1.ZIP › Primary Data/Primary Data/Figure5/b.tif]

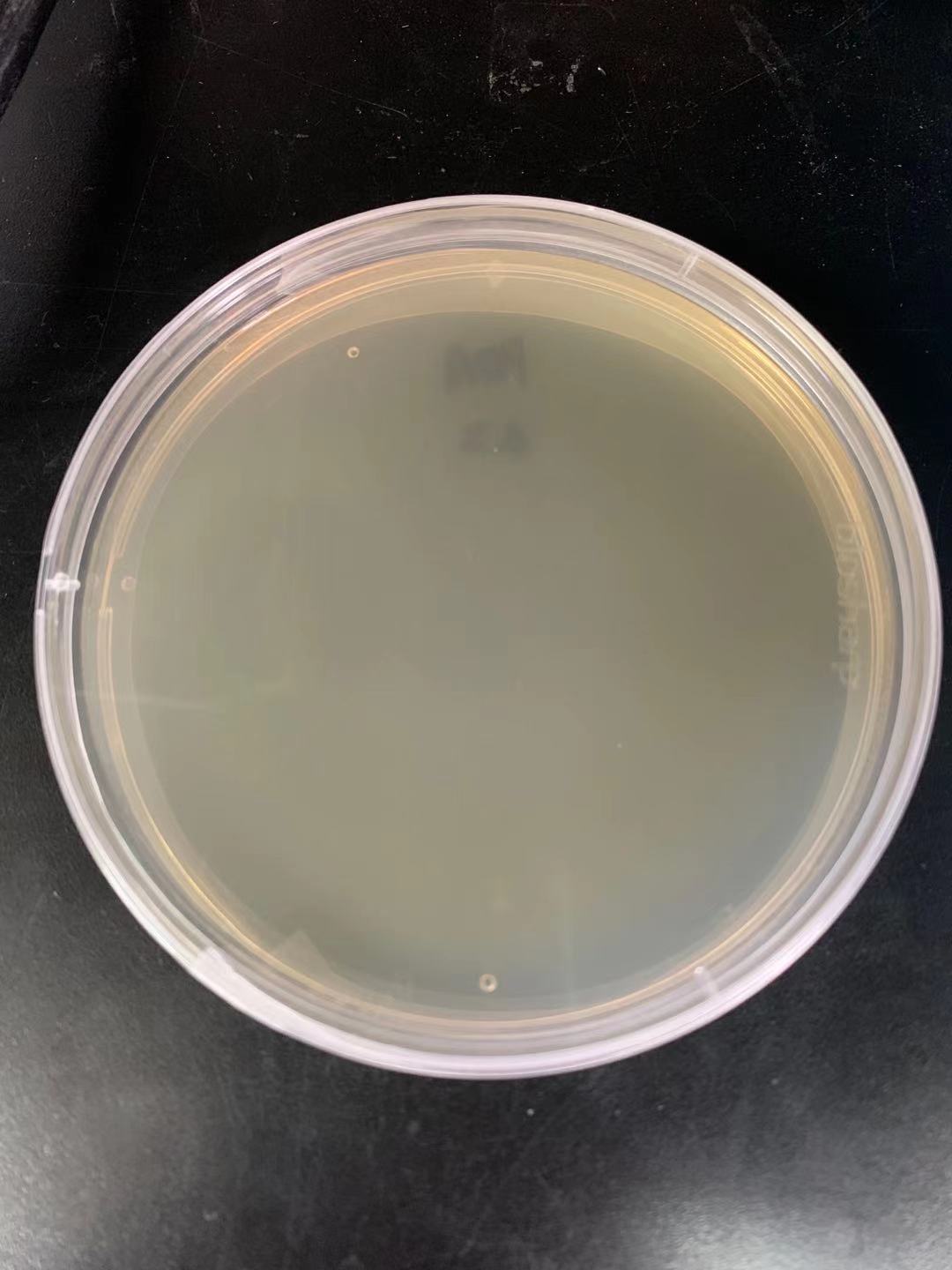

Supplement: Supplementary file 1 [file Data_Sheet_1.ZIP › Primary Data/Primary Data/Figure6/3PDA.jpg]

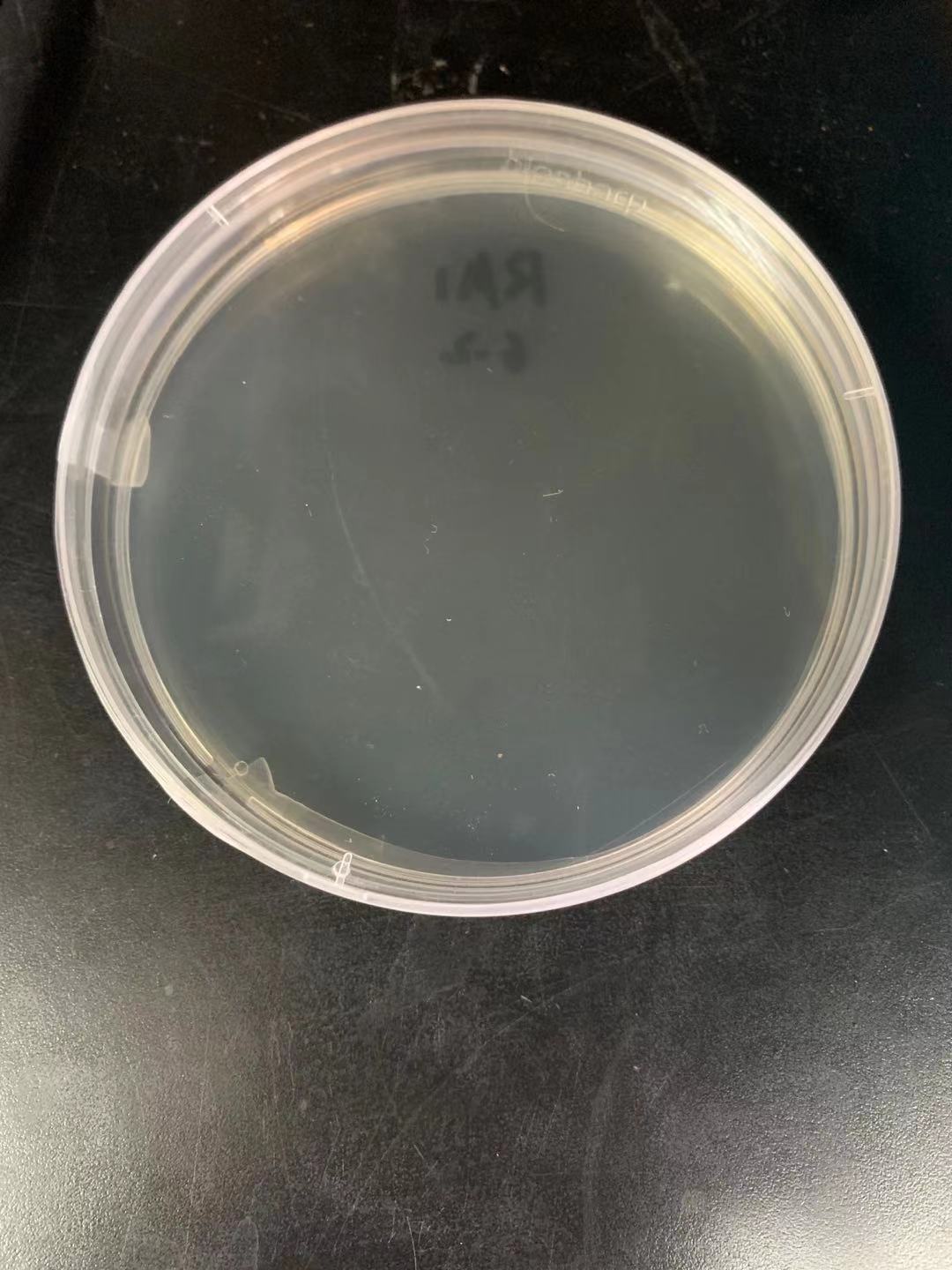

Supplement: Supplementary file 1 [file Data_Sheet_1.ZIP › Primary Data/Primary Data/Figure6/3RM.jpg]

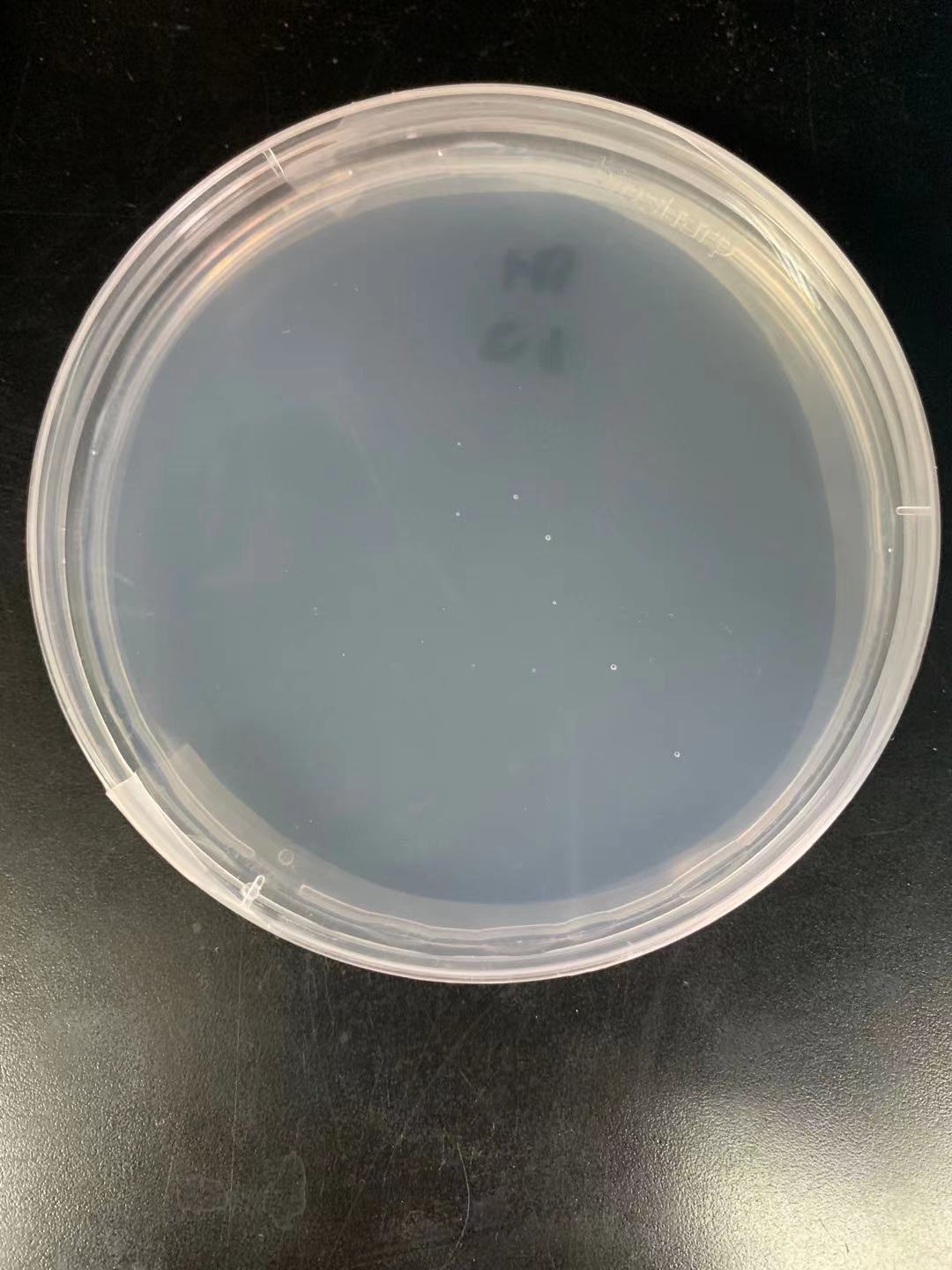

Supplement: Supplementary file 1 [file Data_Sheet_1.ZIP › Primary Data/Primary Data/Figure6/3SH.jpg]

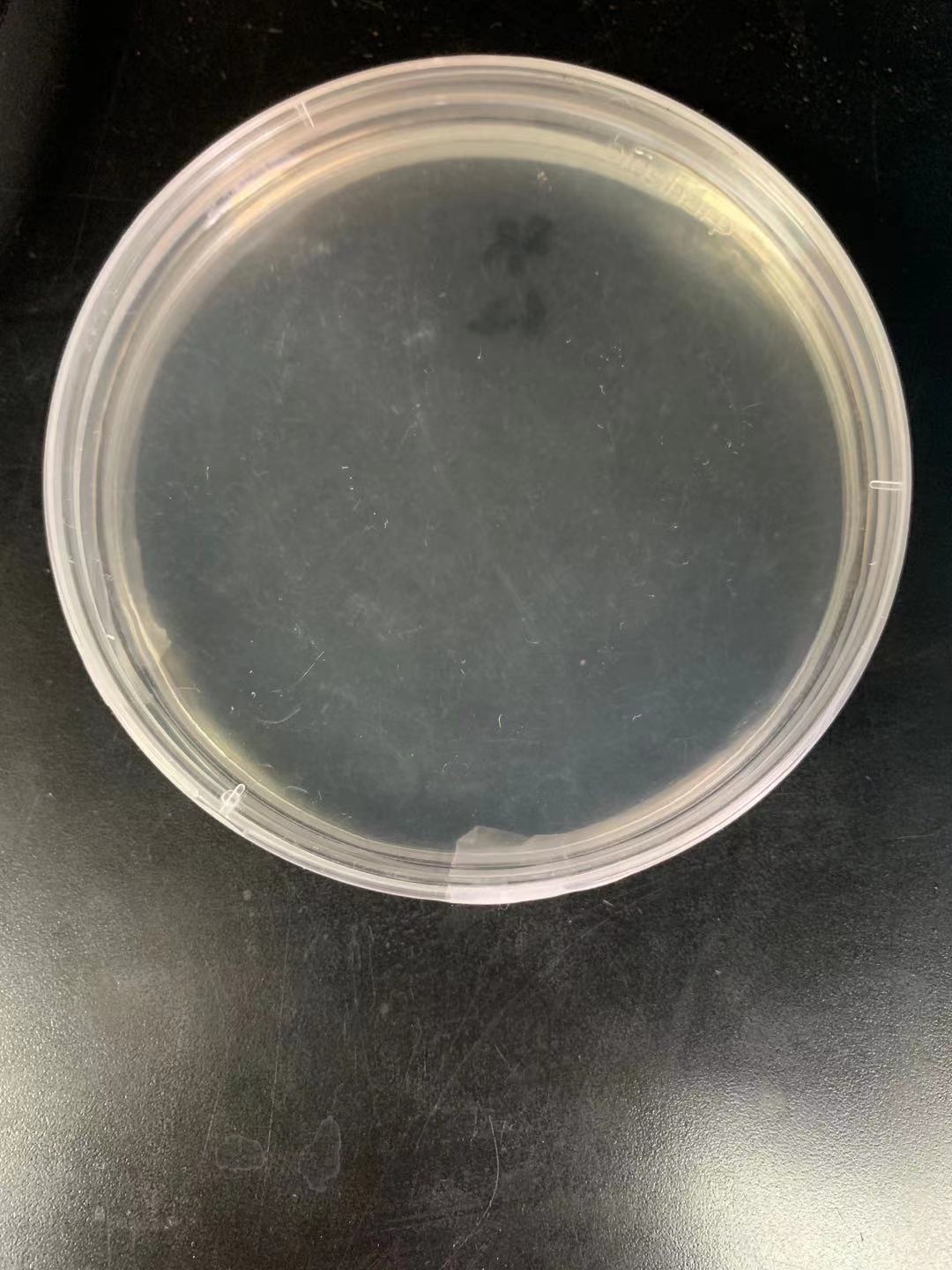

Supplement: Supplementary file 1 [file Data_Sheet_1.ZIP › Primary Data/Primary Data/Figure6/3SR.jpg]

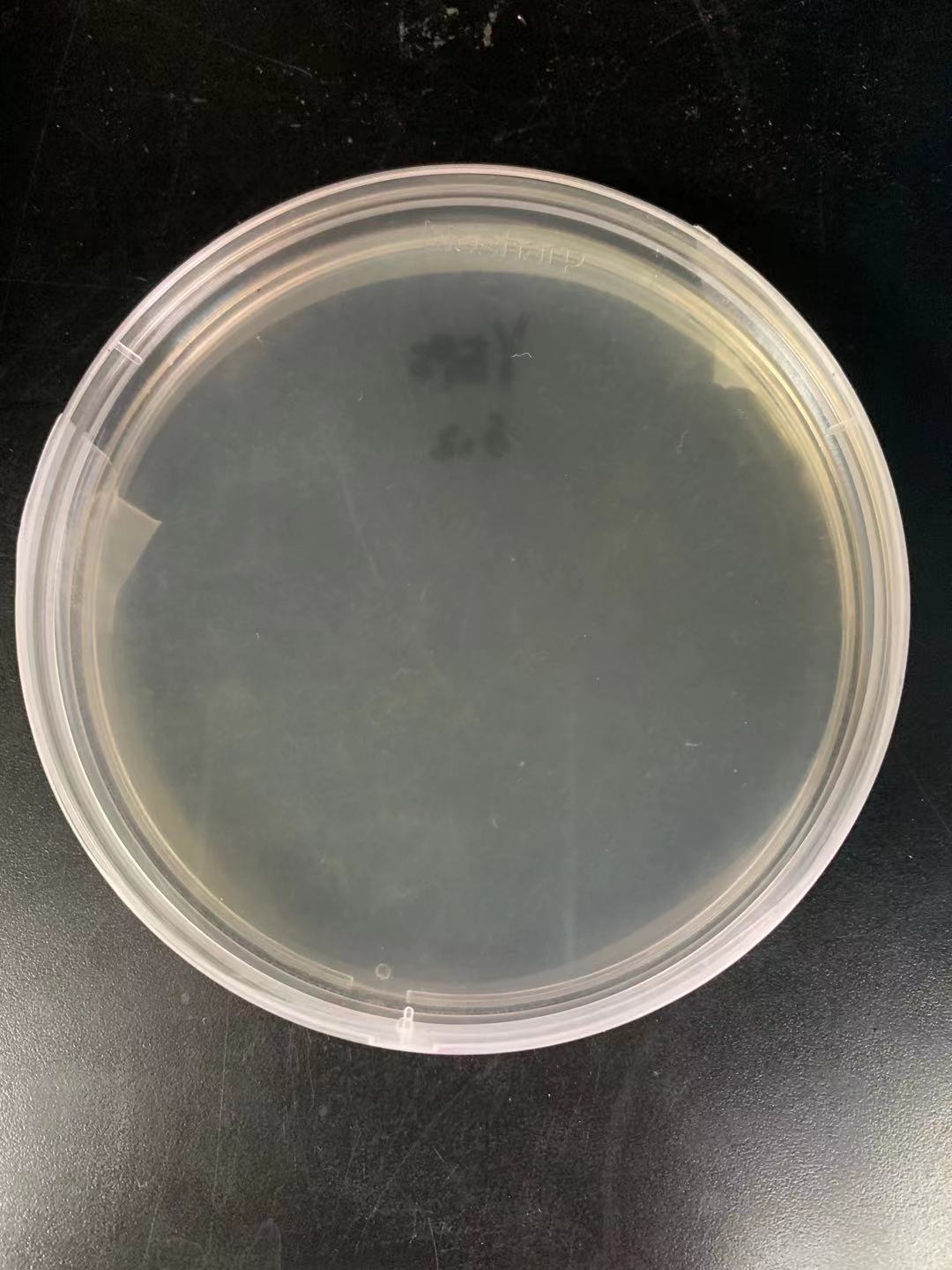

Supplement: Supplementary file 1 [file Data_Sheet_1.ZIP › Primary Data/Primary Data/Figure6/3YEPS.jpg]

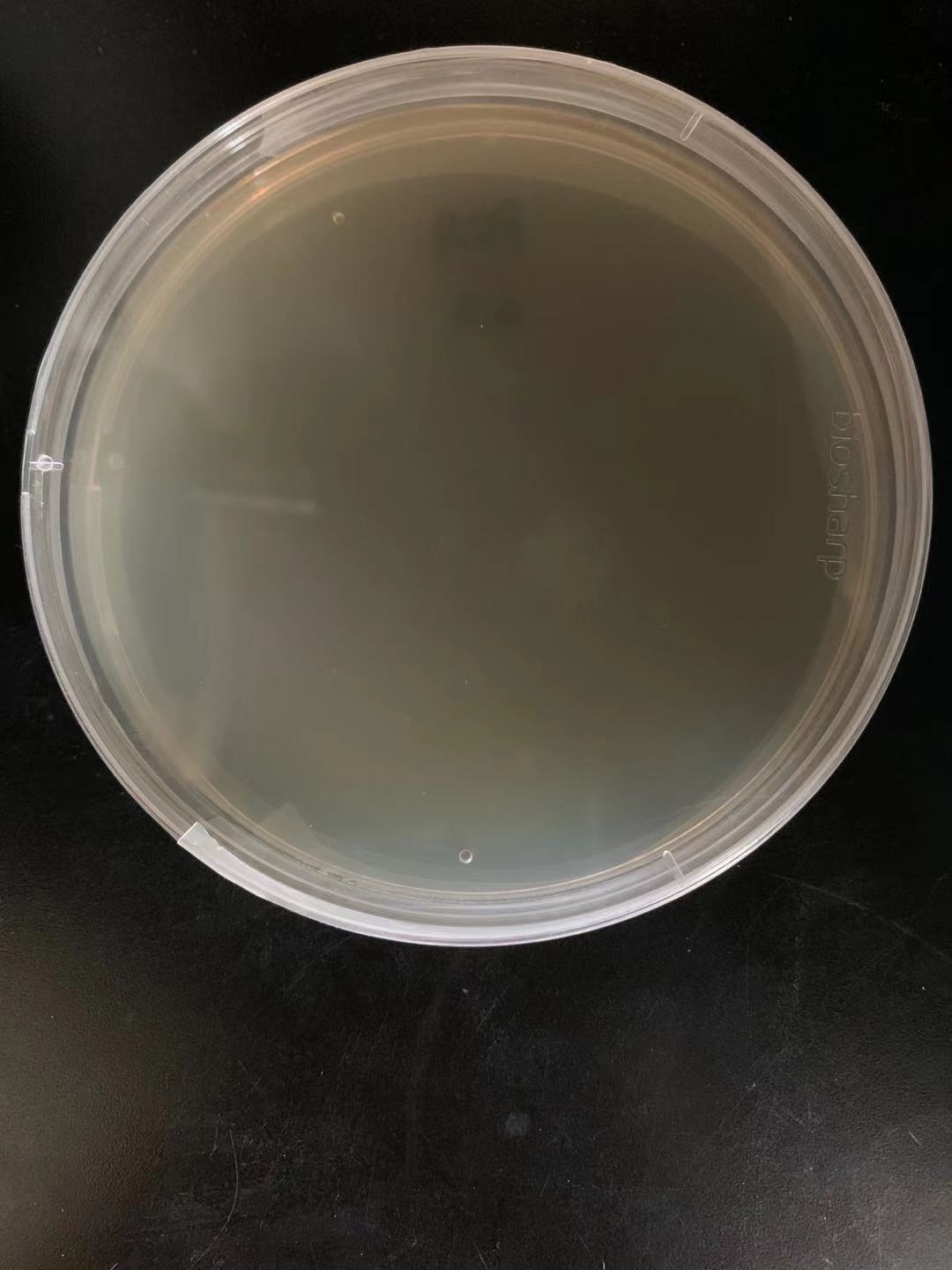

Supplement: Supplementary file 1 [file Data_Sheet_1.ZIP › Primary Data/Primary Data/Figure6/4PDA.jpg]

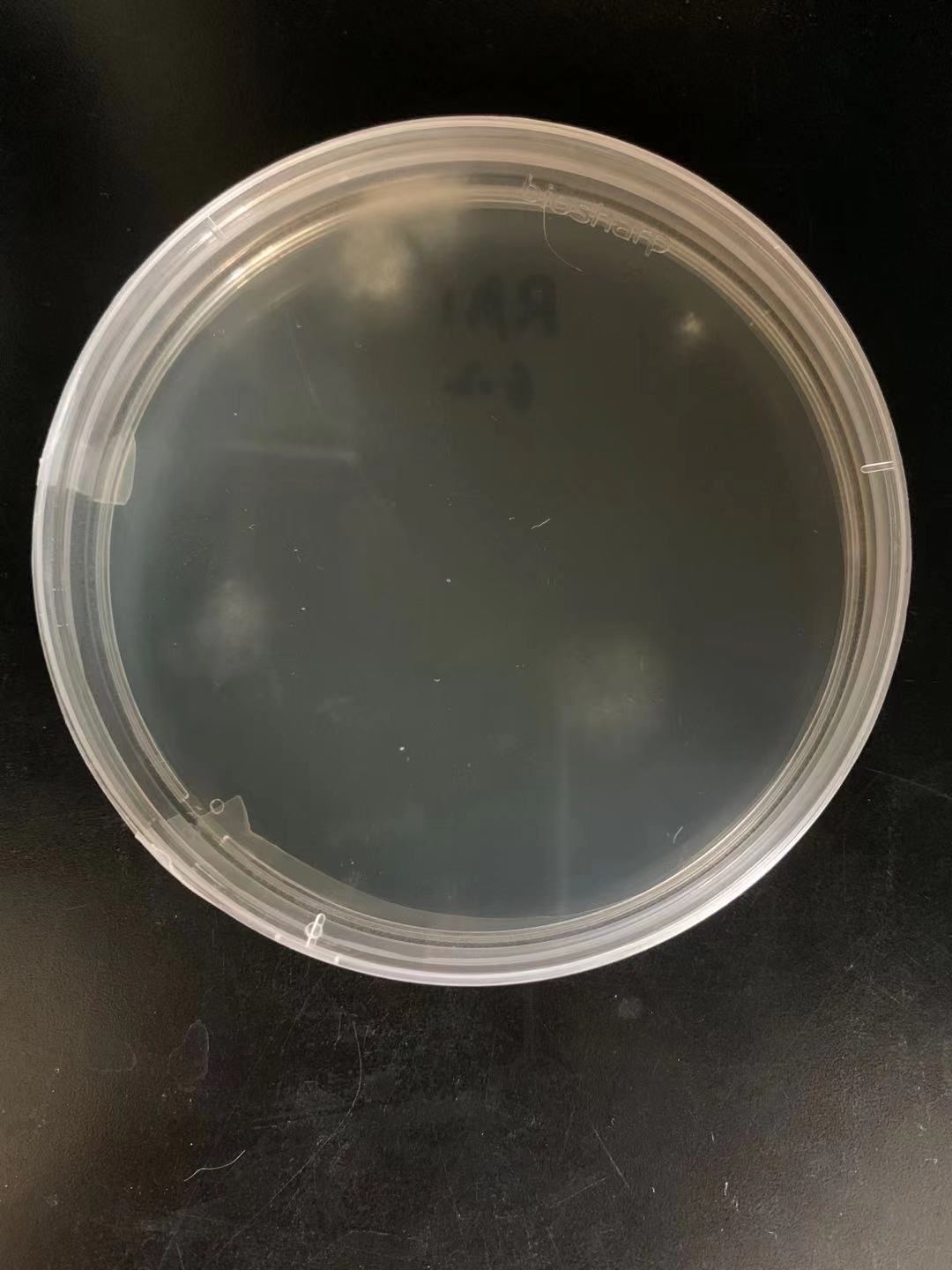

Supplement: Supplementary file 1 [file Data_Sheet_1.ZIP › Primary Data/Primary Data/Figure6/4RM.jpg]

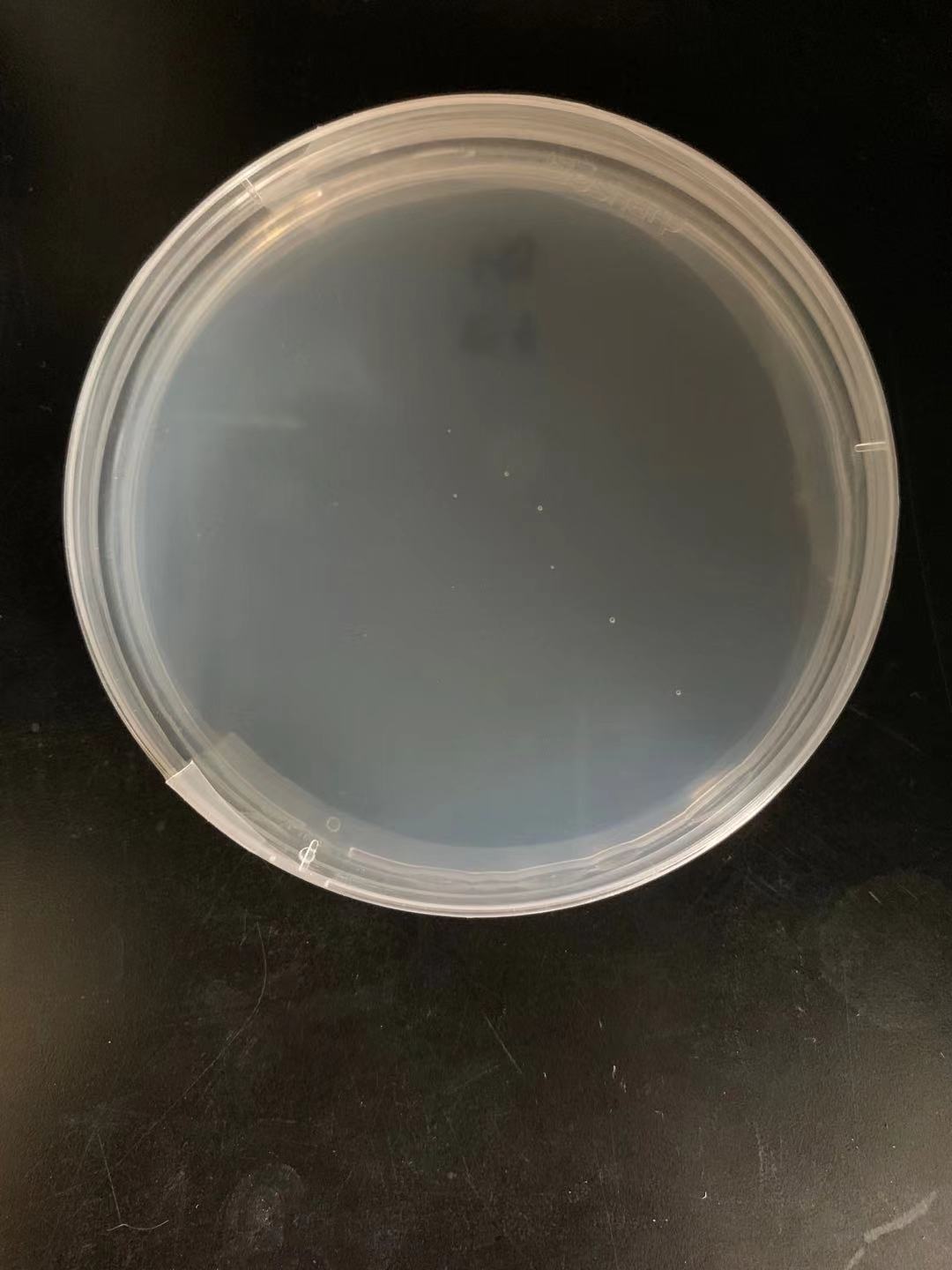

Supplement: Supplementary file 1 [file Data_Sheet_1.ZIP › Primary Data/Primary Data/Figure6/4SH.jpg]

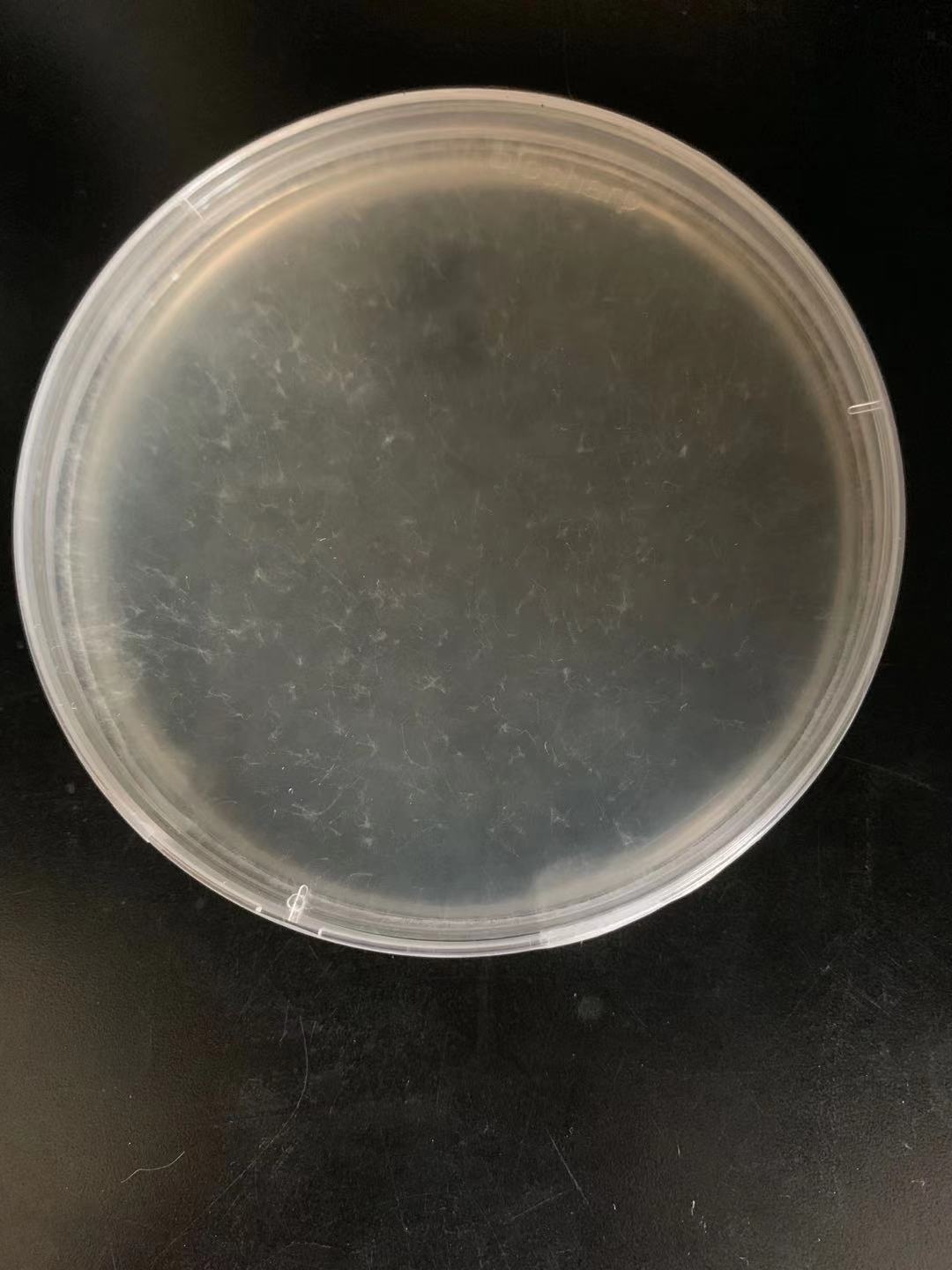

Supplement: Supplementary file 1 [file Data_Sheet_1.ZIP › Primary Data/Primary Data/Figure6/4SR.jpg]

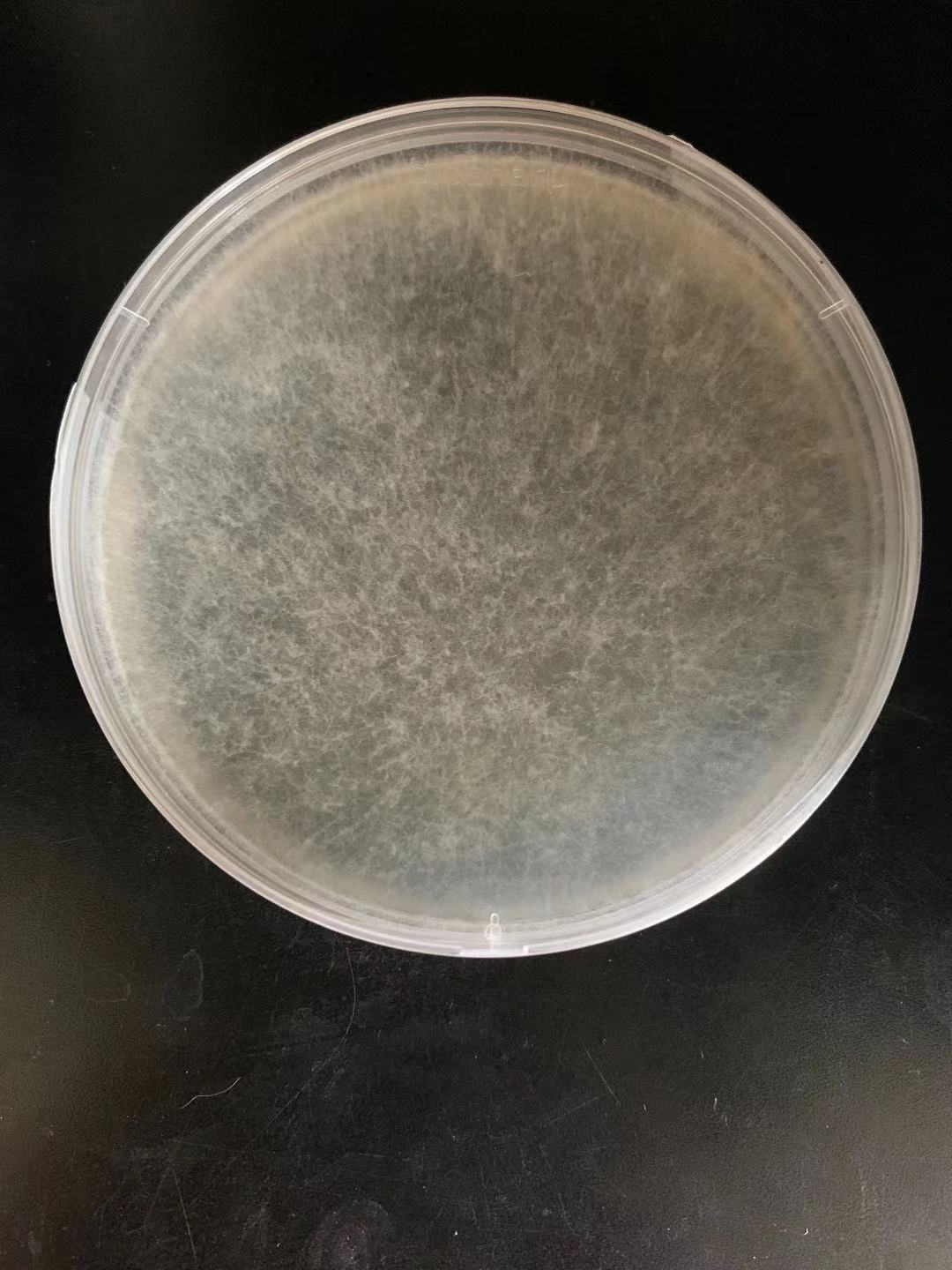

Supplement: Supplementary file 1 [file Data_Sheet_1.ZIP › Primary Data/Primary Data/Figure6/4YEPS.jpg]

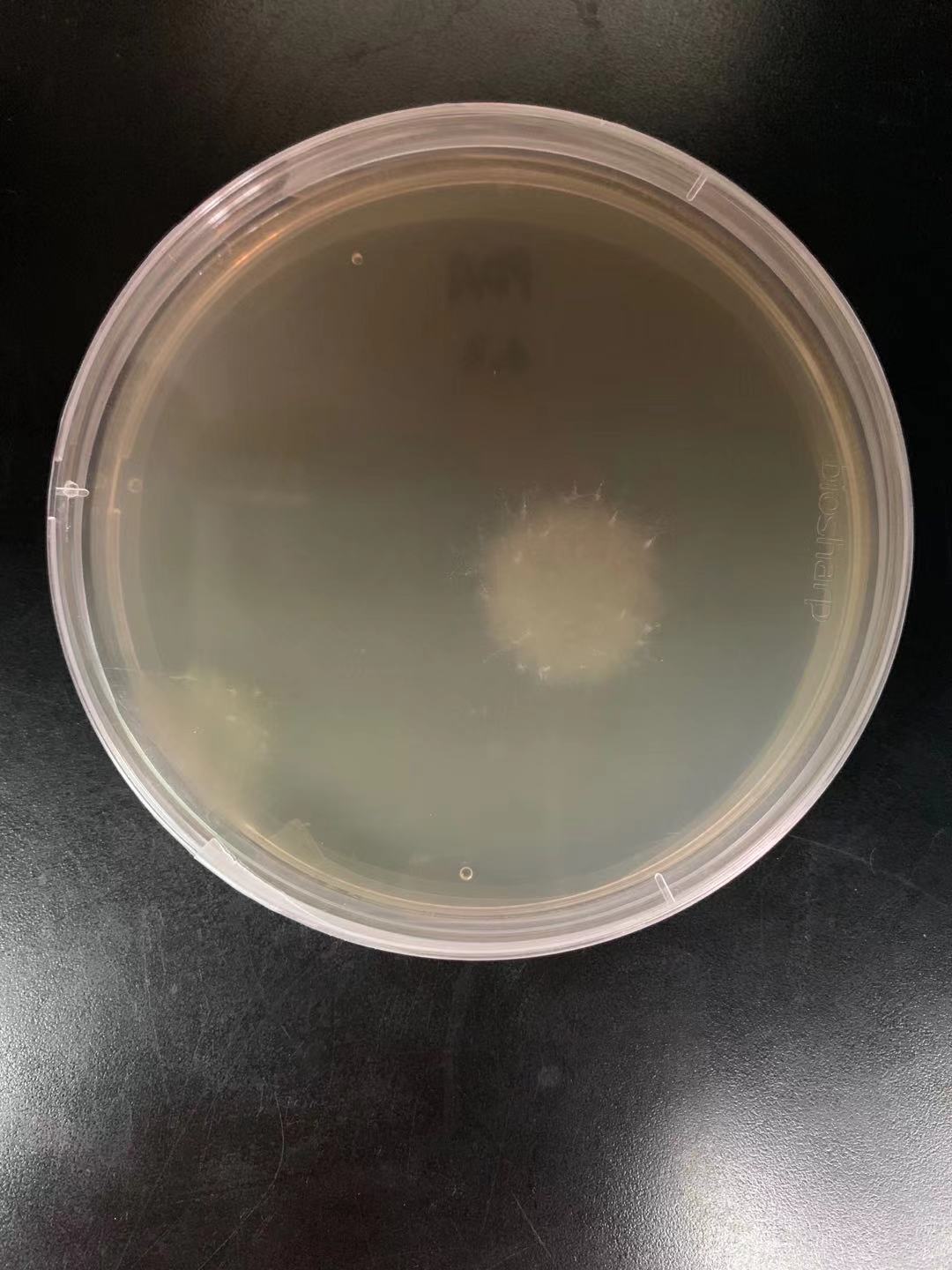

Supplement: Supplementary file 1 [file Data_Sheet_1.ZIP › Primary Data/Primary Data/Figure6/5PDA.jpg]

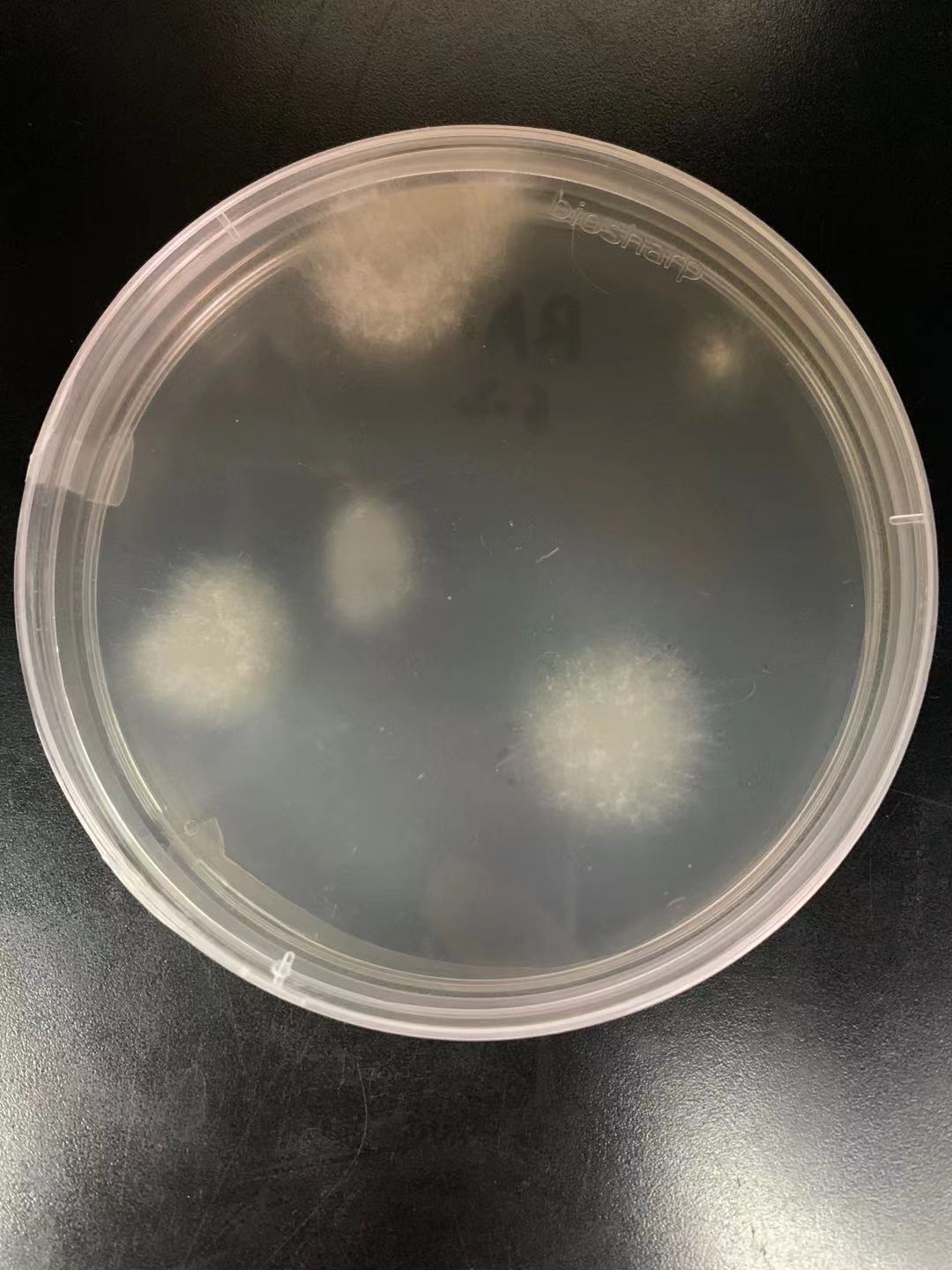

Supplement: Supplementary file 1 [file Data_Sheet_1.ZIP › Primary Data/Primary Data/Figure6/5RM.jpg]

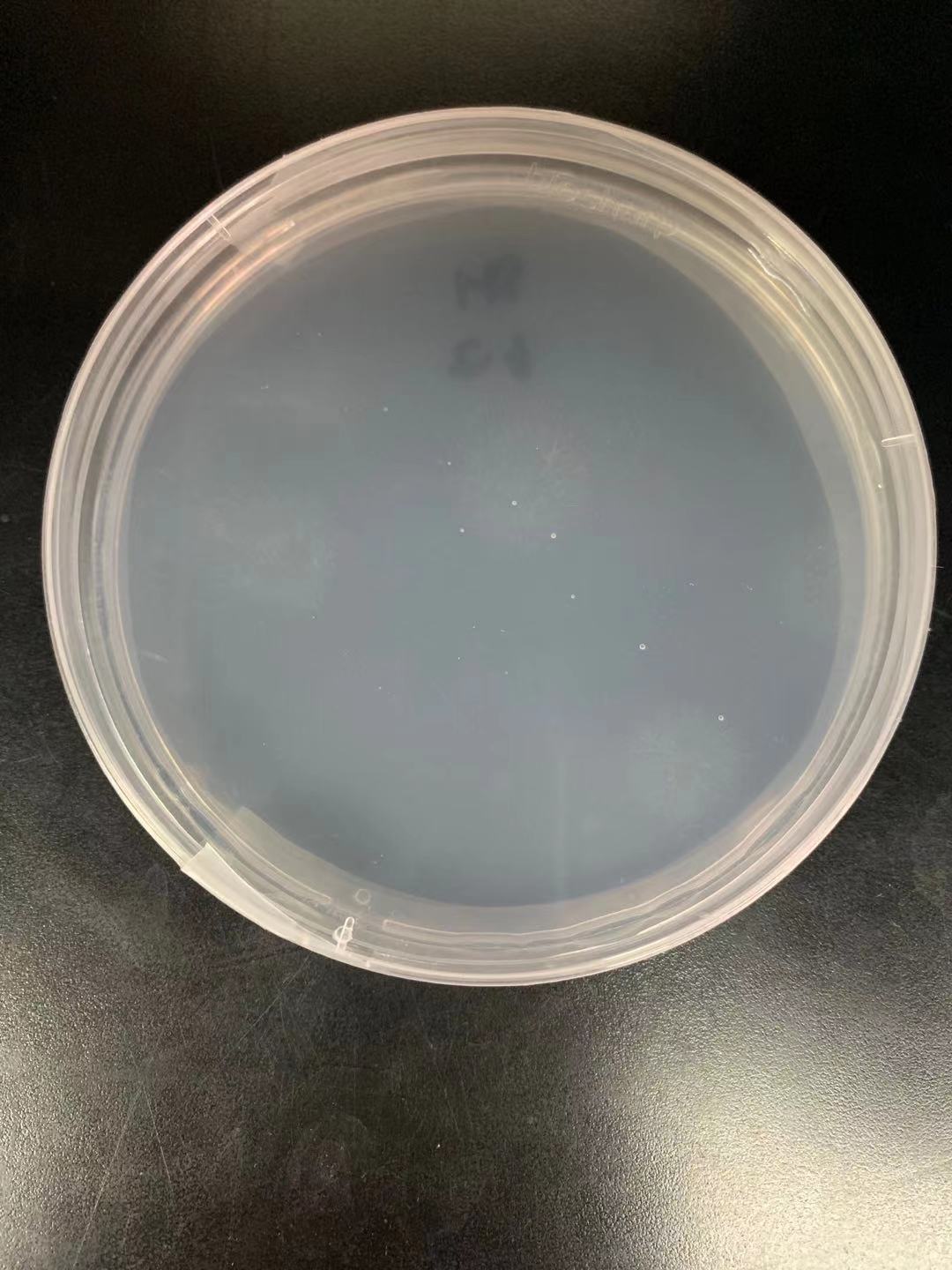

Supplement: Supplementary file 1 [file Data_Sheet_1.ZIP › Primary Data/Primary Data/Figure6/5SH.jpg]

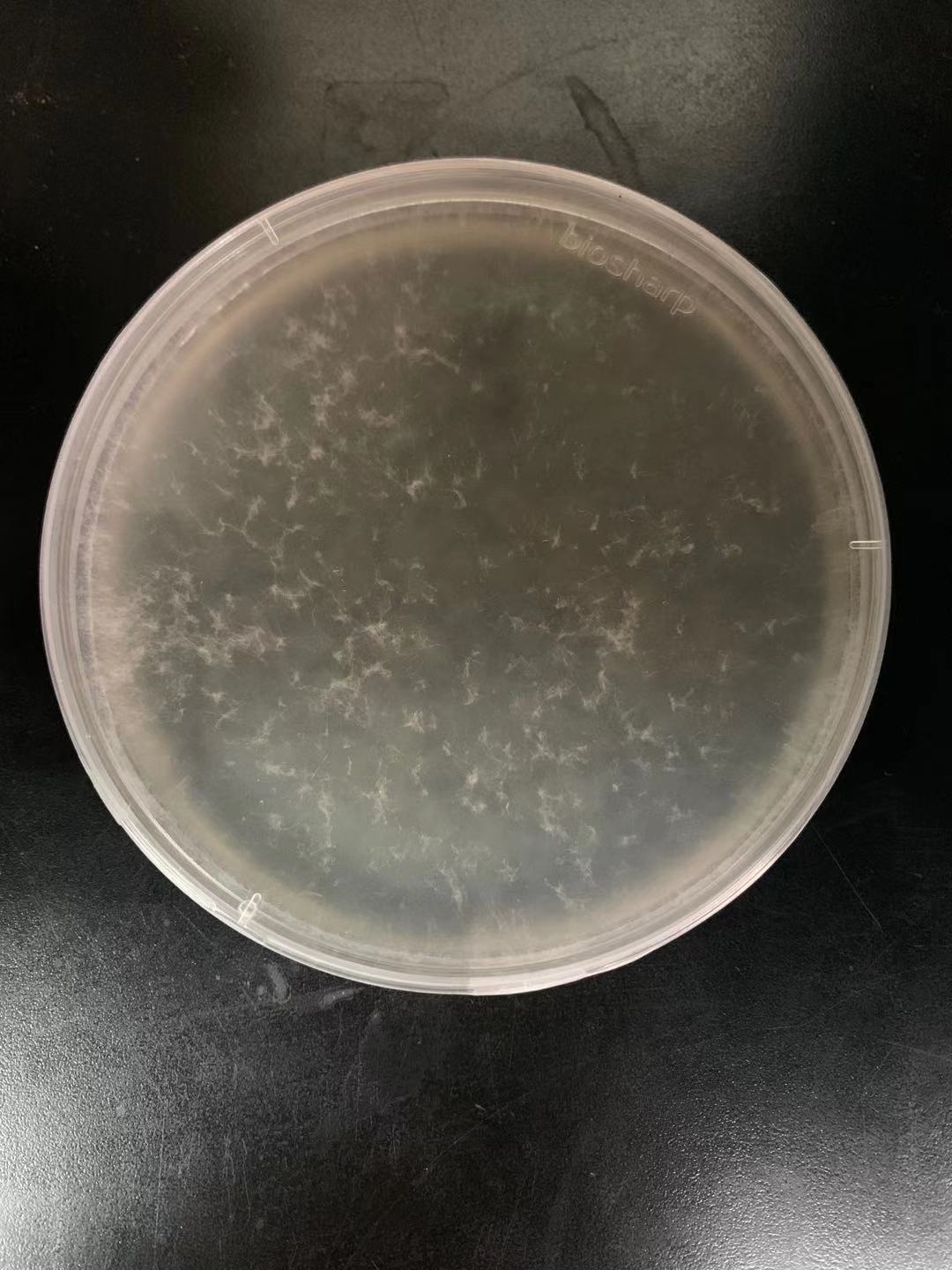

Supplement: Supplementary file 1 [file Data_Sheet_1.ZIP › Primary Data/Primary Data/Figure6/5SR.jpg]

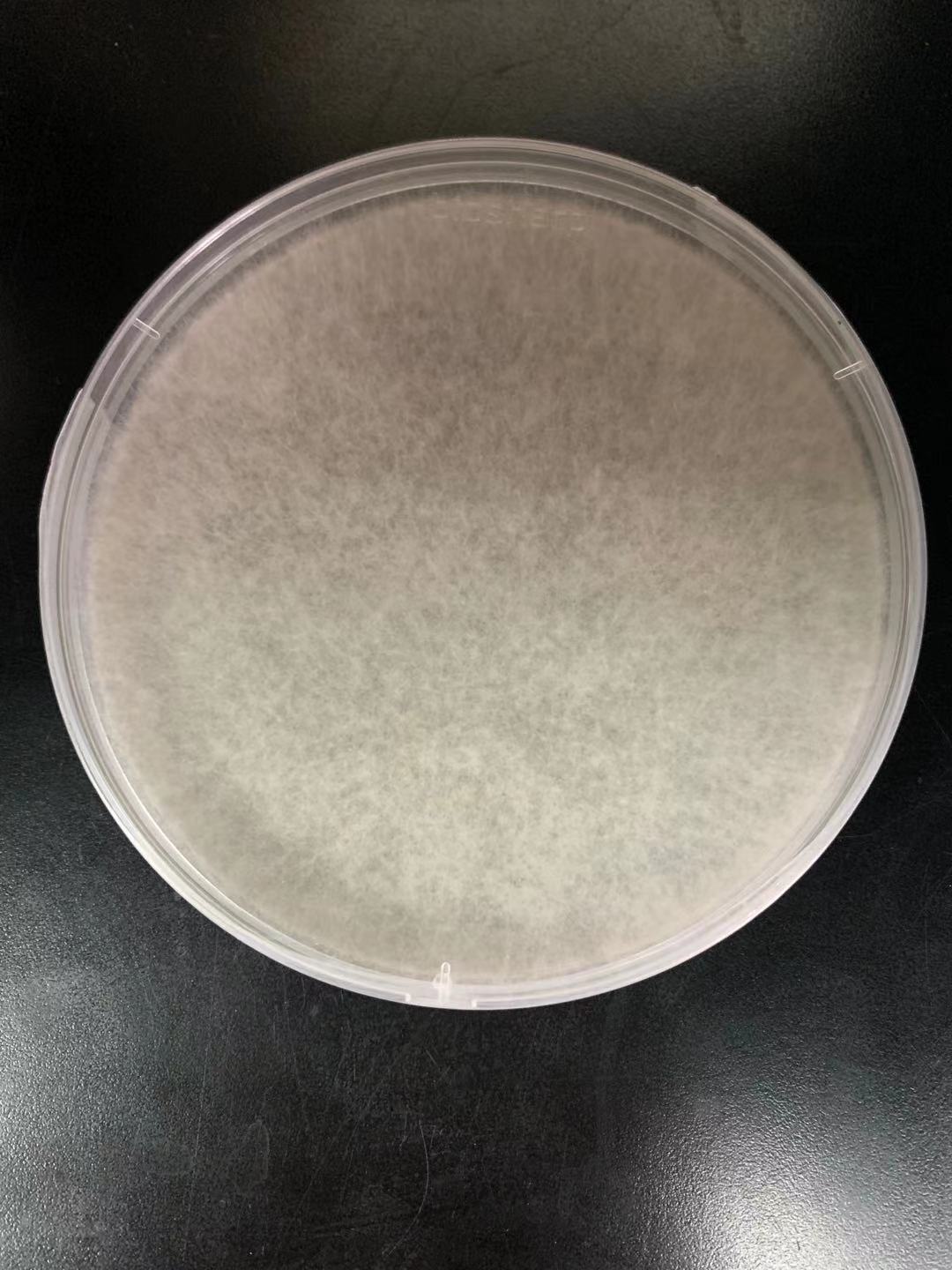

Supplement: Supplementary file 1 [file Data_Sheet_1.ZIP › Primary Data/Primary Data/Figure6/5YEPS.jpg]

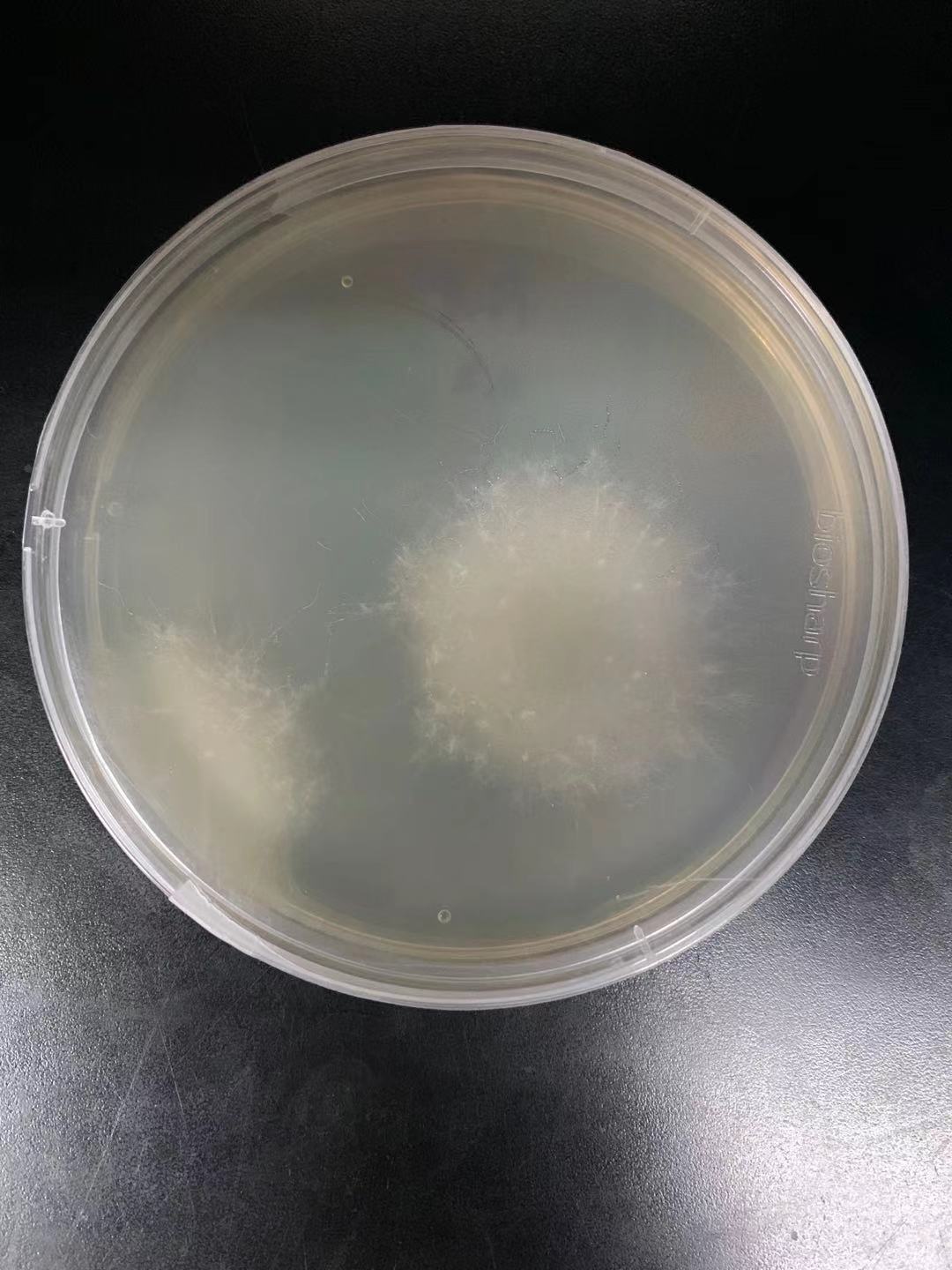

Supplement: Supplementary file 1 [file Data_Sheet_1.ZIP › Primary Data/Primary Data/Figure6/6PDA.jpg]

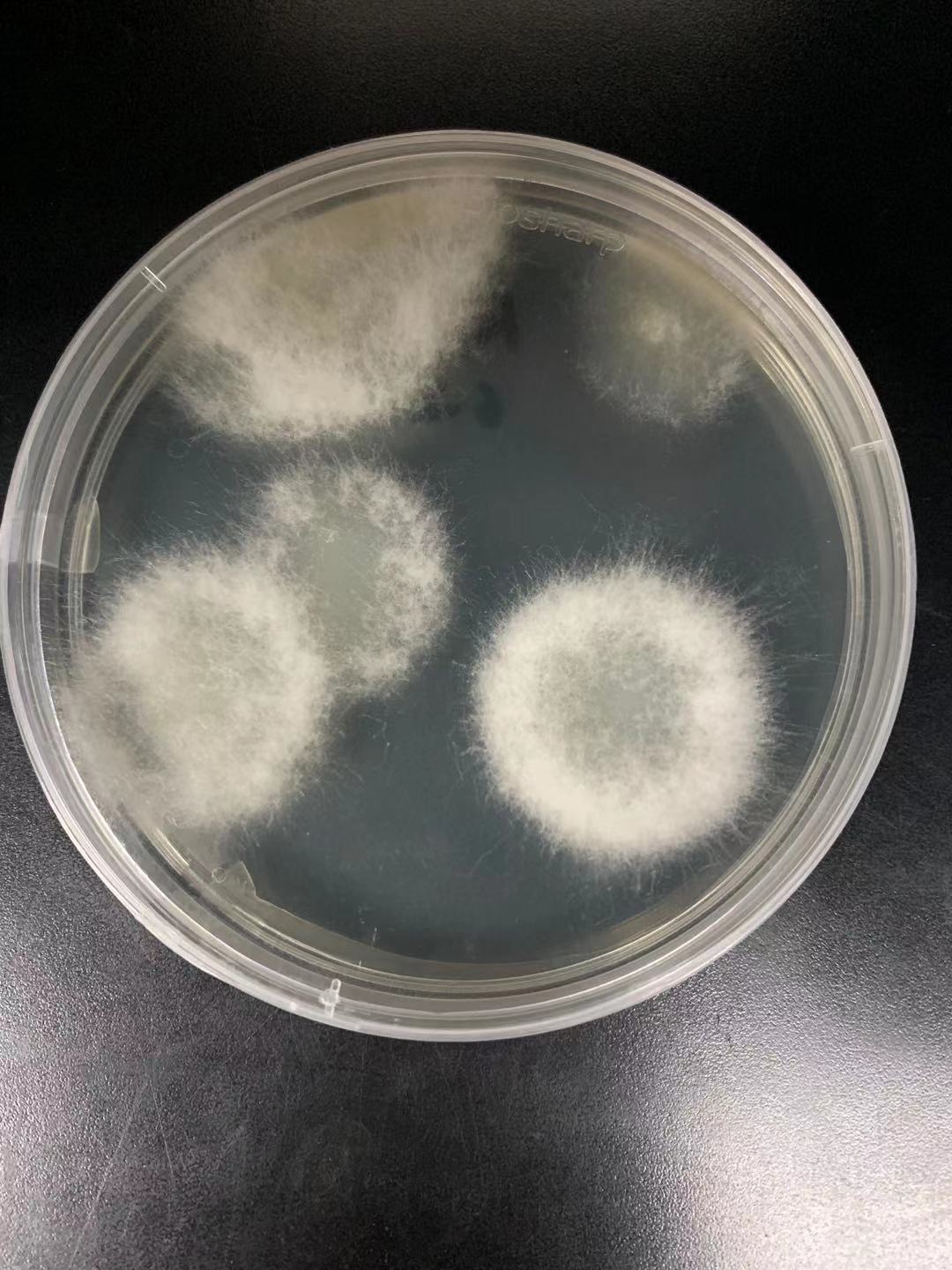

Supplement: Supplementary file 1 [file Data_Sheet_1.ZIP › Primary Data/Primary Data/Figure6/6RM.jpg]

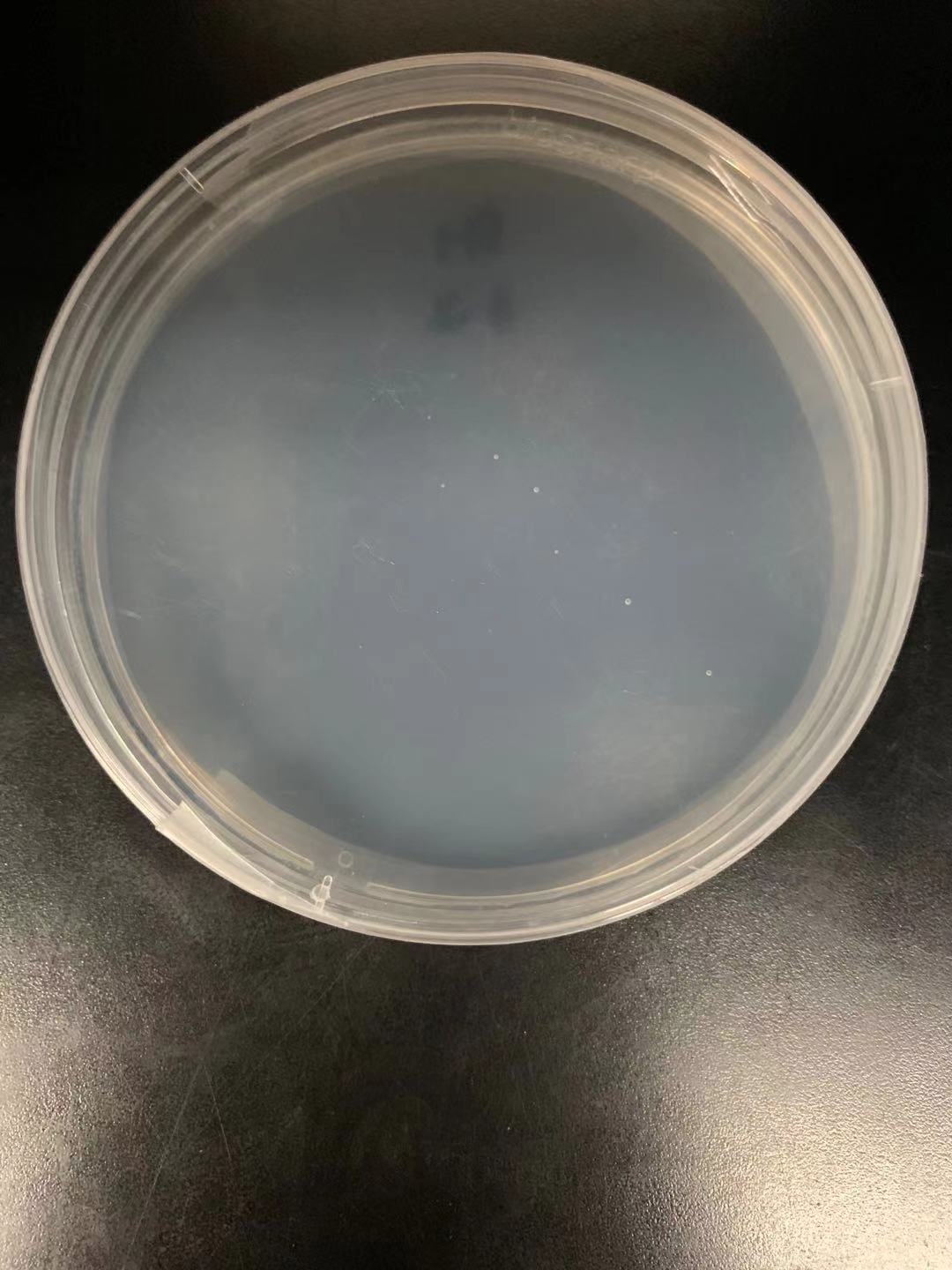

Supplement: Supplementary file 1 [file Data_Sheet_1.ZIP › Primary Data/Primary Data/Figure6/6SH.jpg]

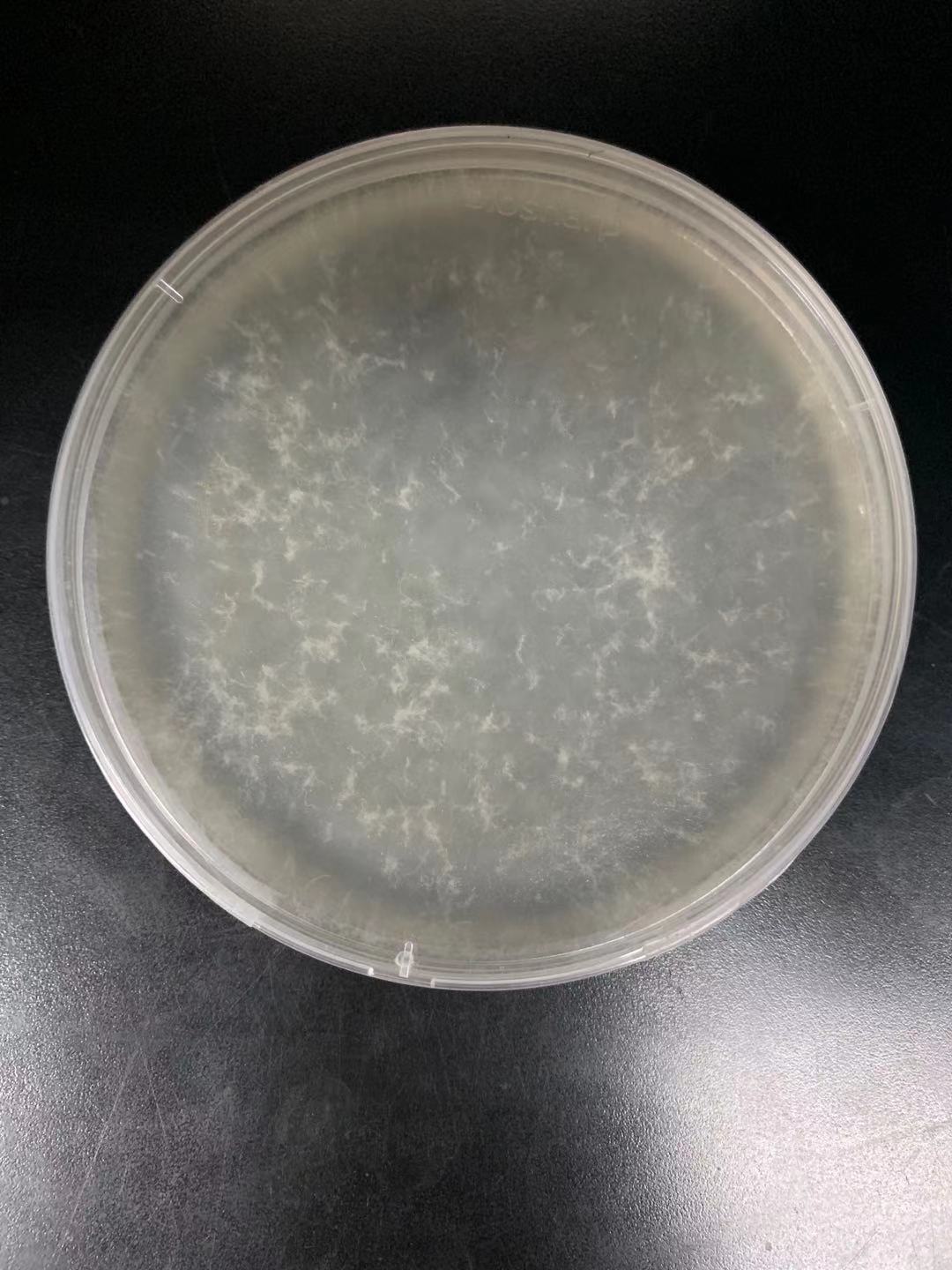

Supplement: Supplementary file 1 [file Data_Sheet_1.ZIP › Primary Data/Primary Data/Figure6/6SR.jpg]

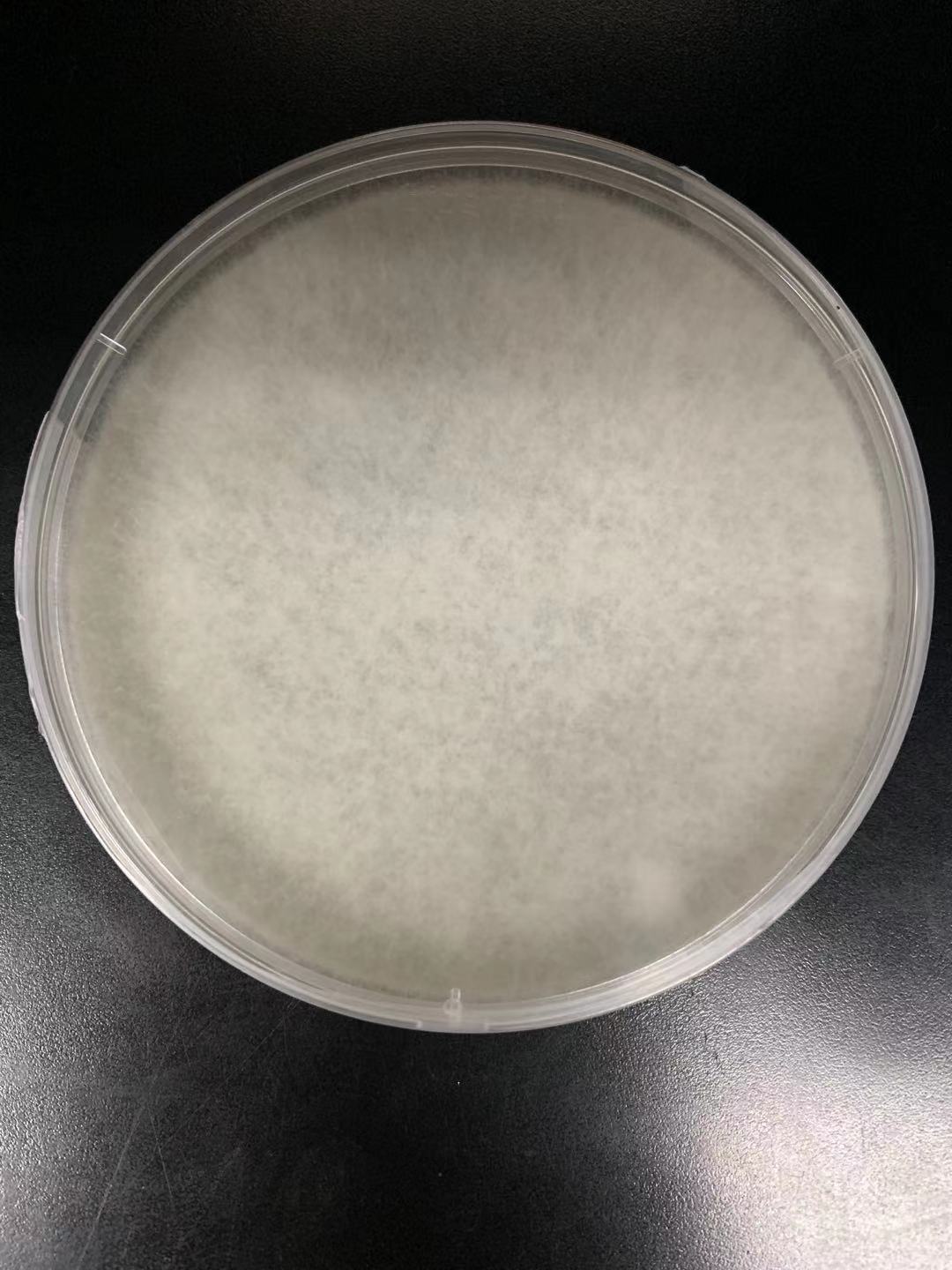

Supplement: Supplementary file 1 [file Data_Sheet_1.ZIP › Primary Data/Primary Data/Figure6/6YEPS.jpg]

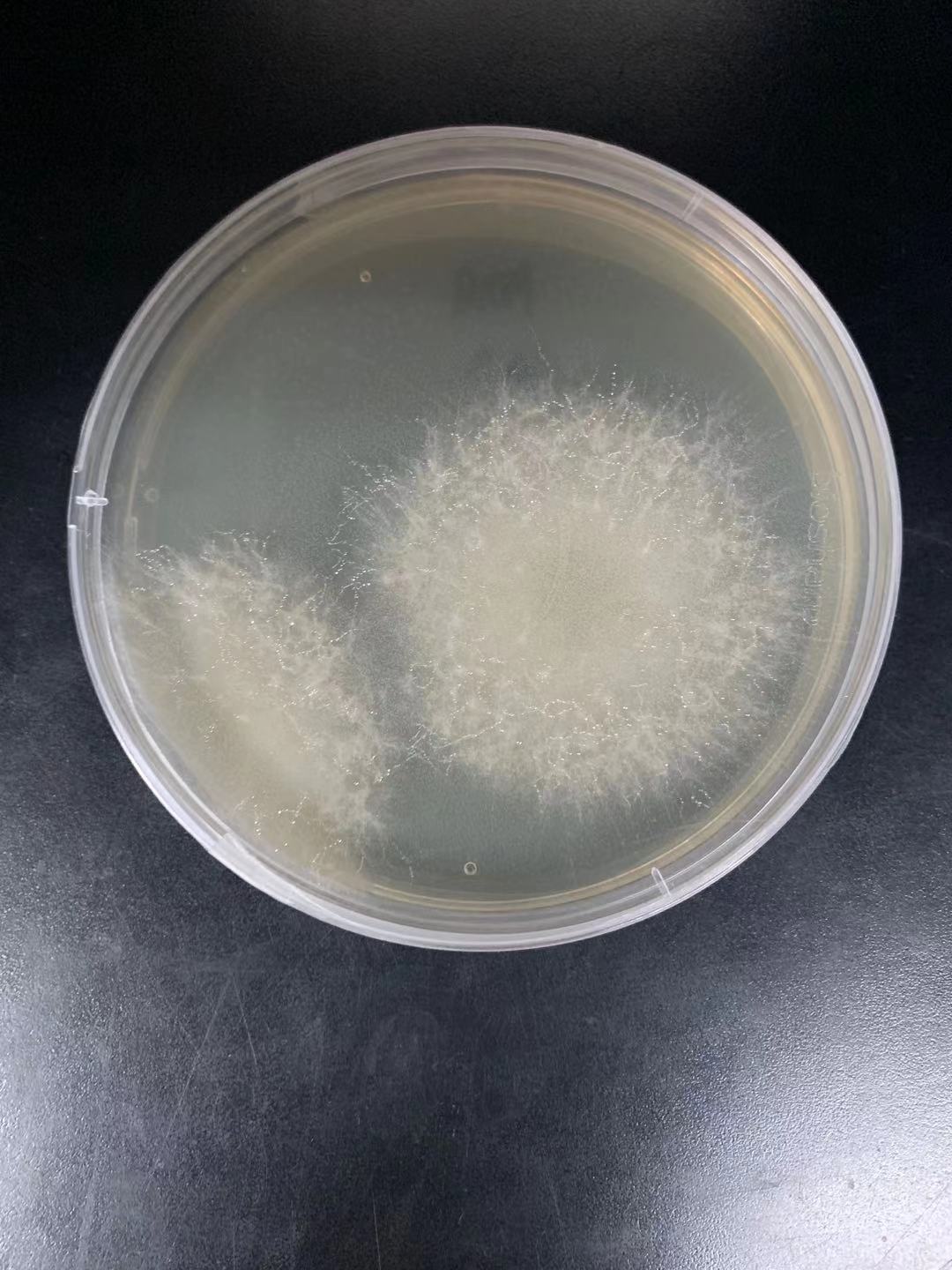

Supplement: Supplementary file 1 [file Data_Sheet_1.ZIP › Primary Data/Primary Data/Figure6/7PDA.jpg]

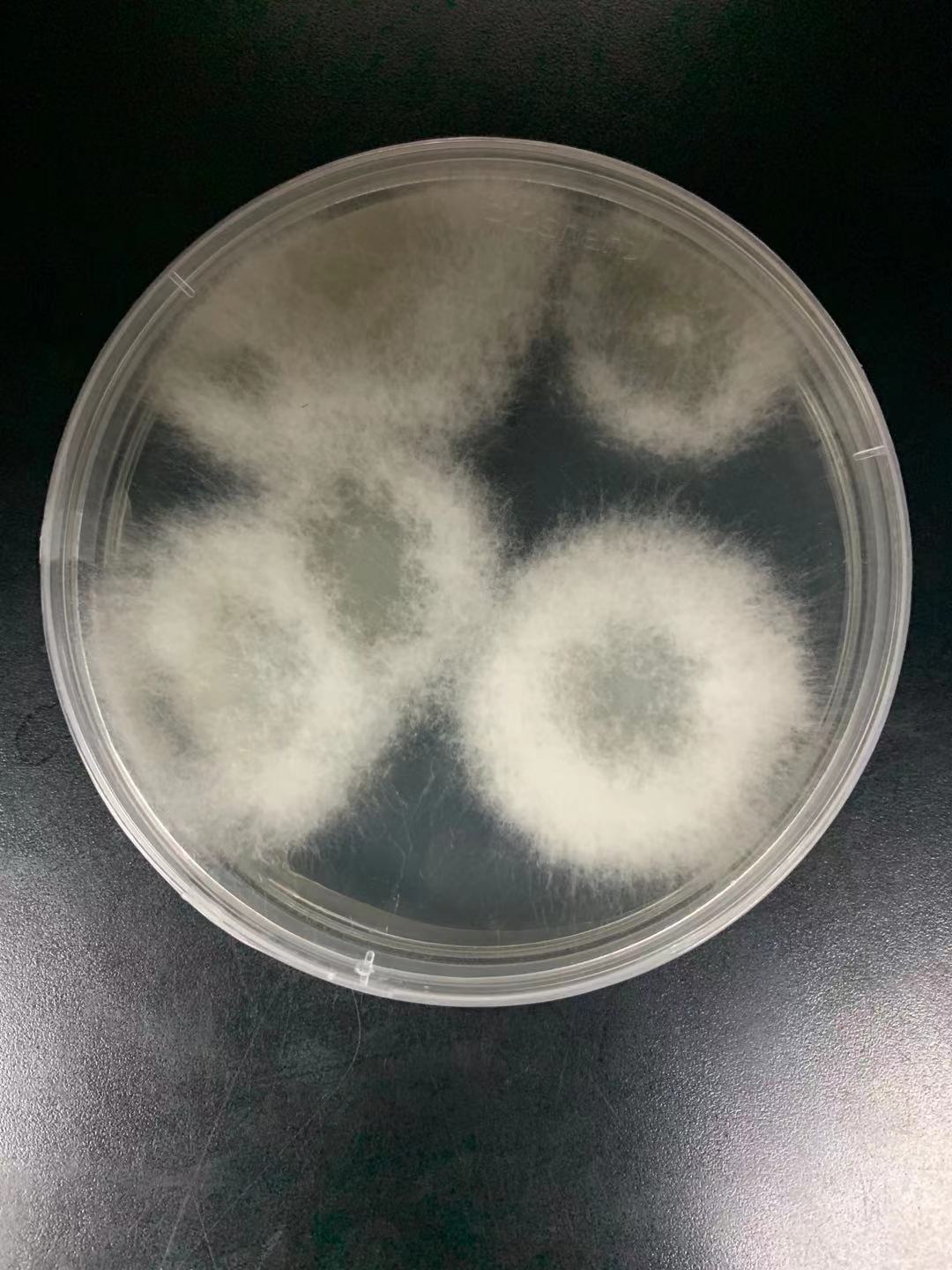

Supplement: Supplementary file 1 [file Data_Sheet_1.ZIP › Primary Data/Primary Data/Figure6/7RM.jpg]

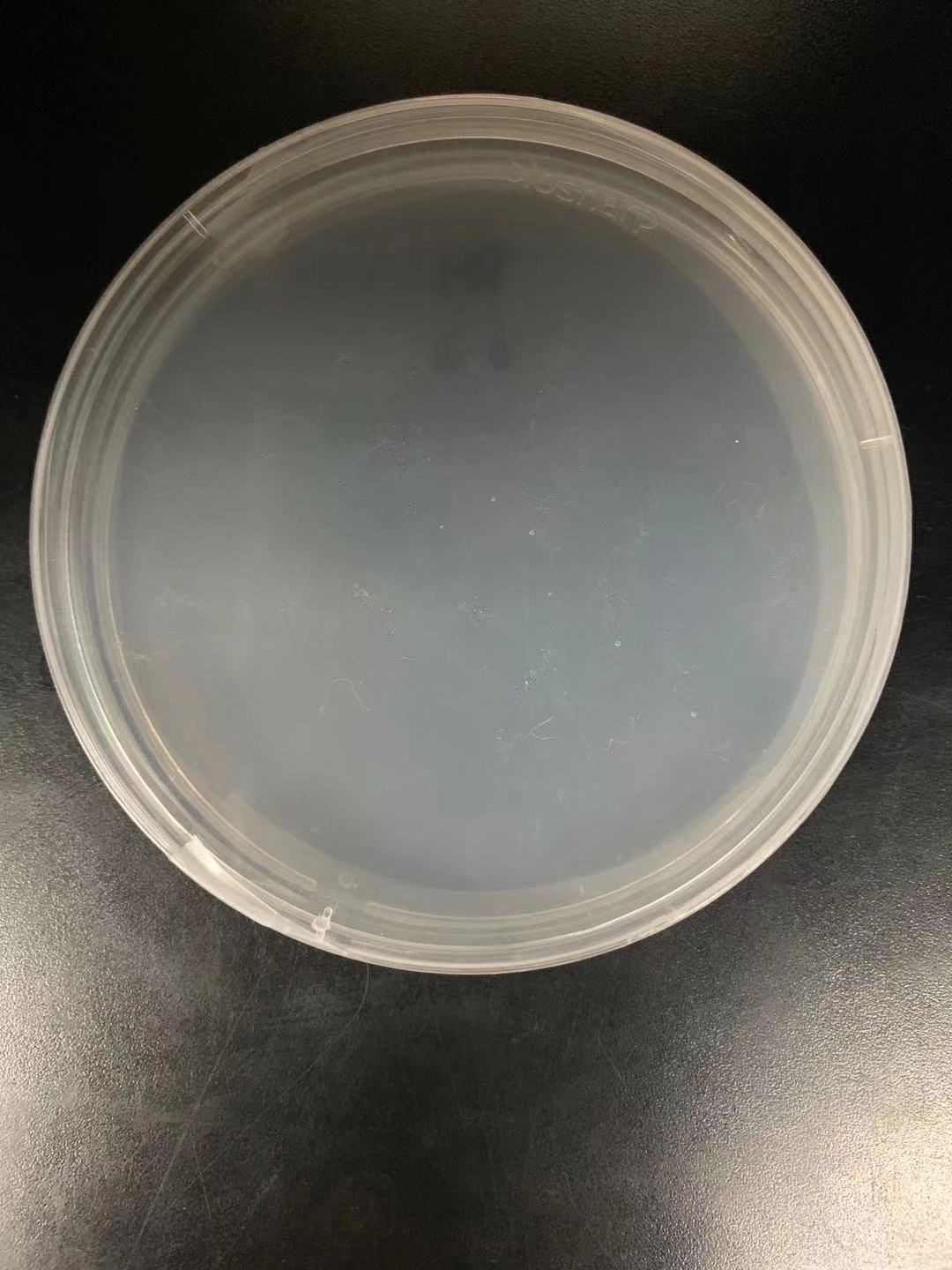

Supplement: Supplementary file 1 [file Data_Sheet_1.ZIP › Primary Data/Primary Data/Figure6/7SH.jpg]

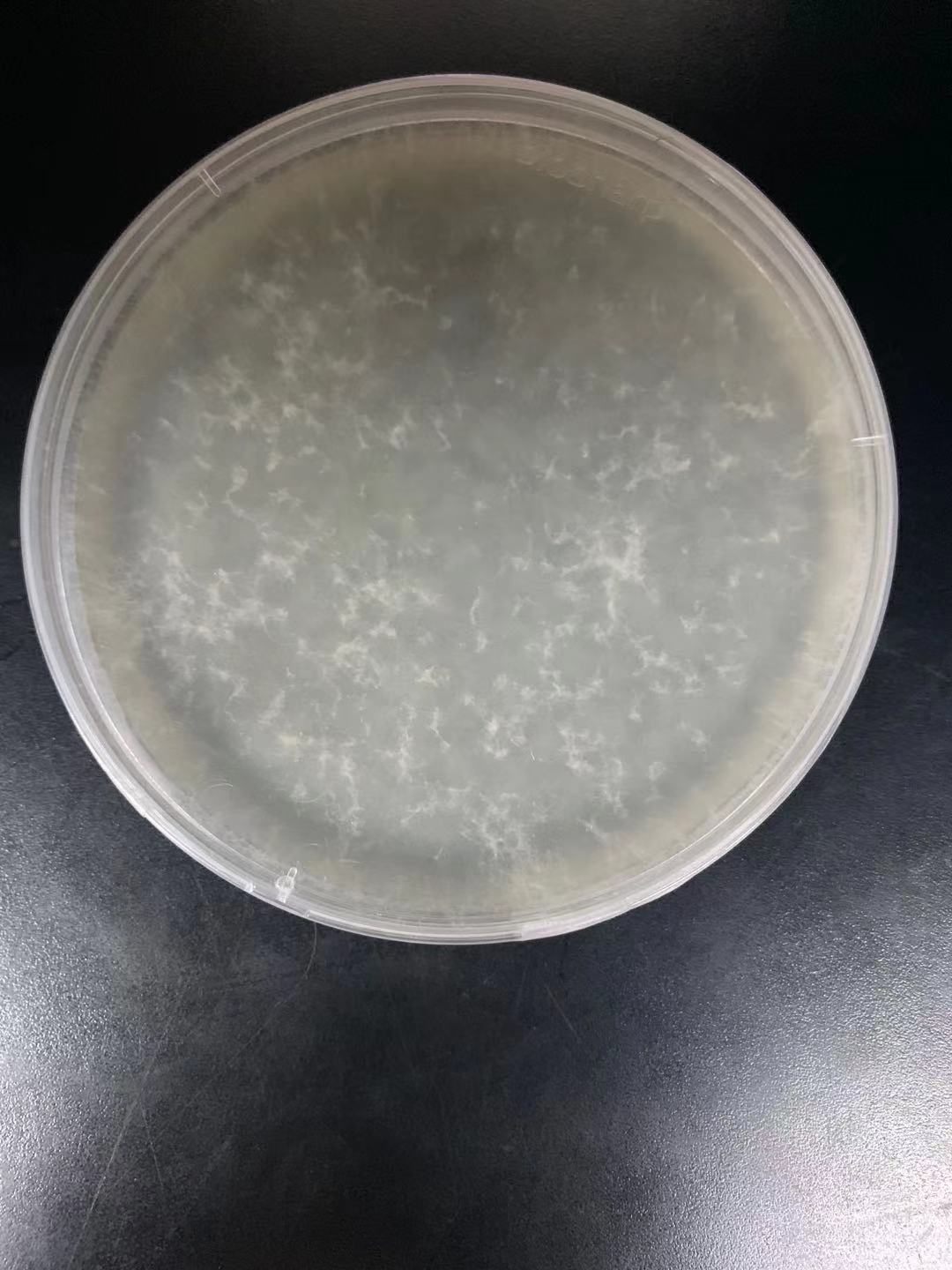

Supplement: Supplementary file 1 [file Data_Sheet_1.ZIP › Primary Data/Primary Data/Figure6/7SR.jpg]

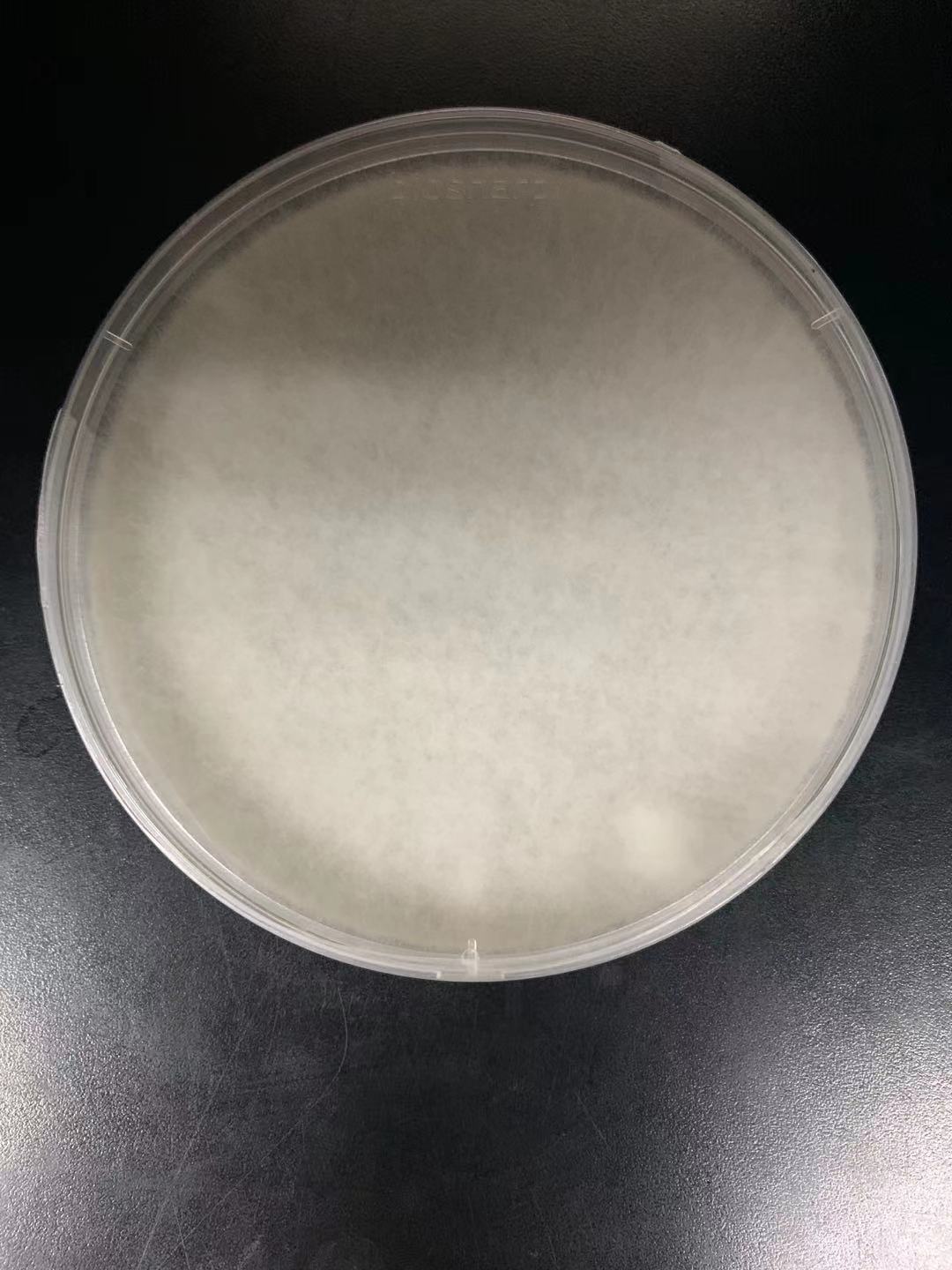

Supplement: Supplementary file 1 [file Data_Sheet_1.ZIP › Primary Data/Primary Data/Figure6/7YEPS.jpg]

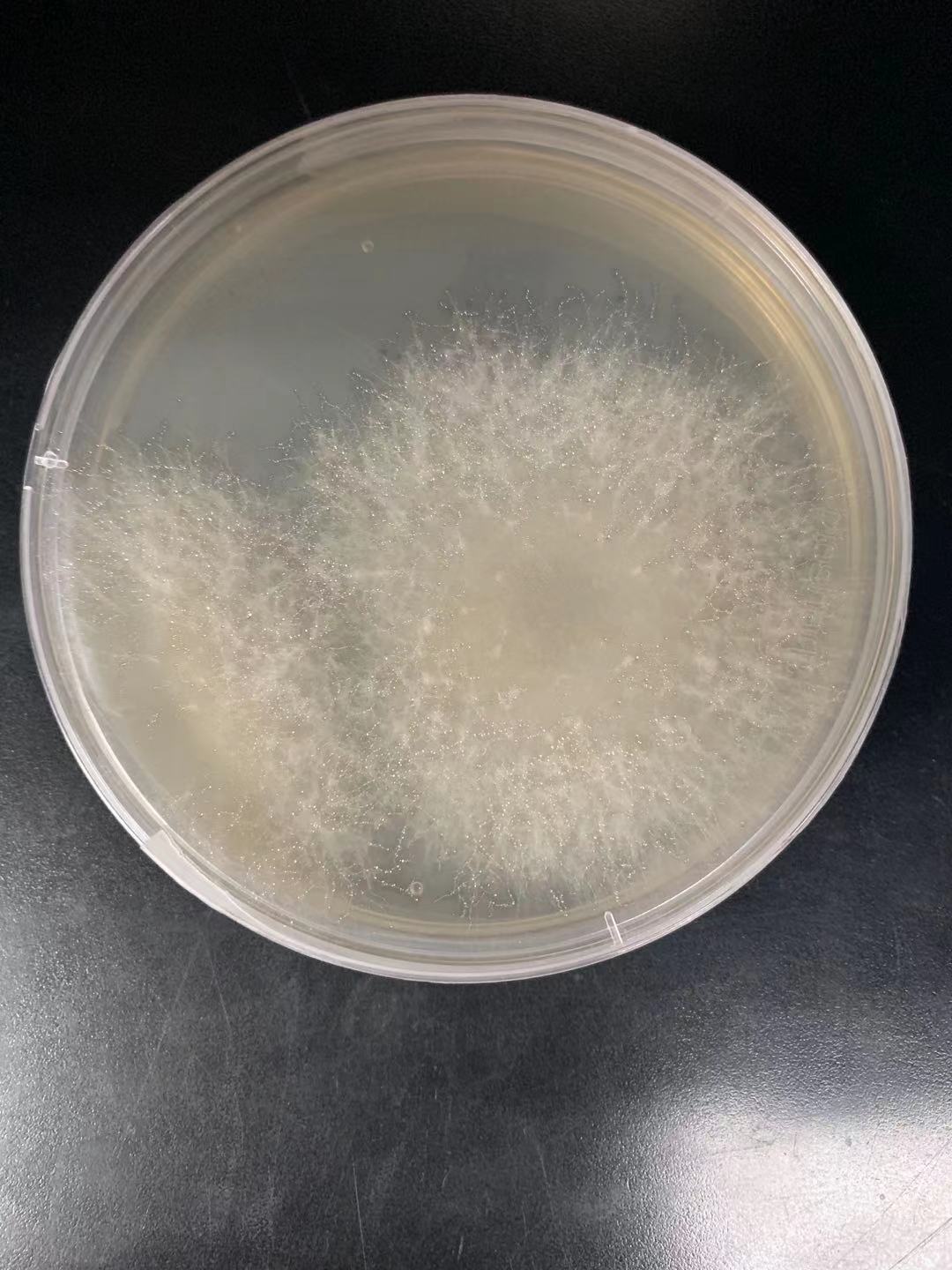

Supplement: Supplementary file 1 [file Data_Sheet_1.ZIP › Primary Data/Primary Data/Figure6/8PDA.jpg]

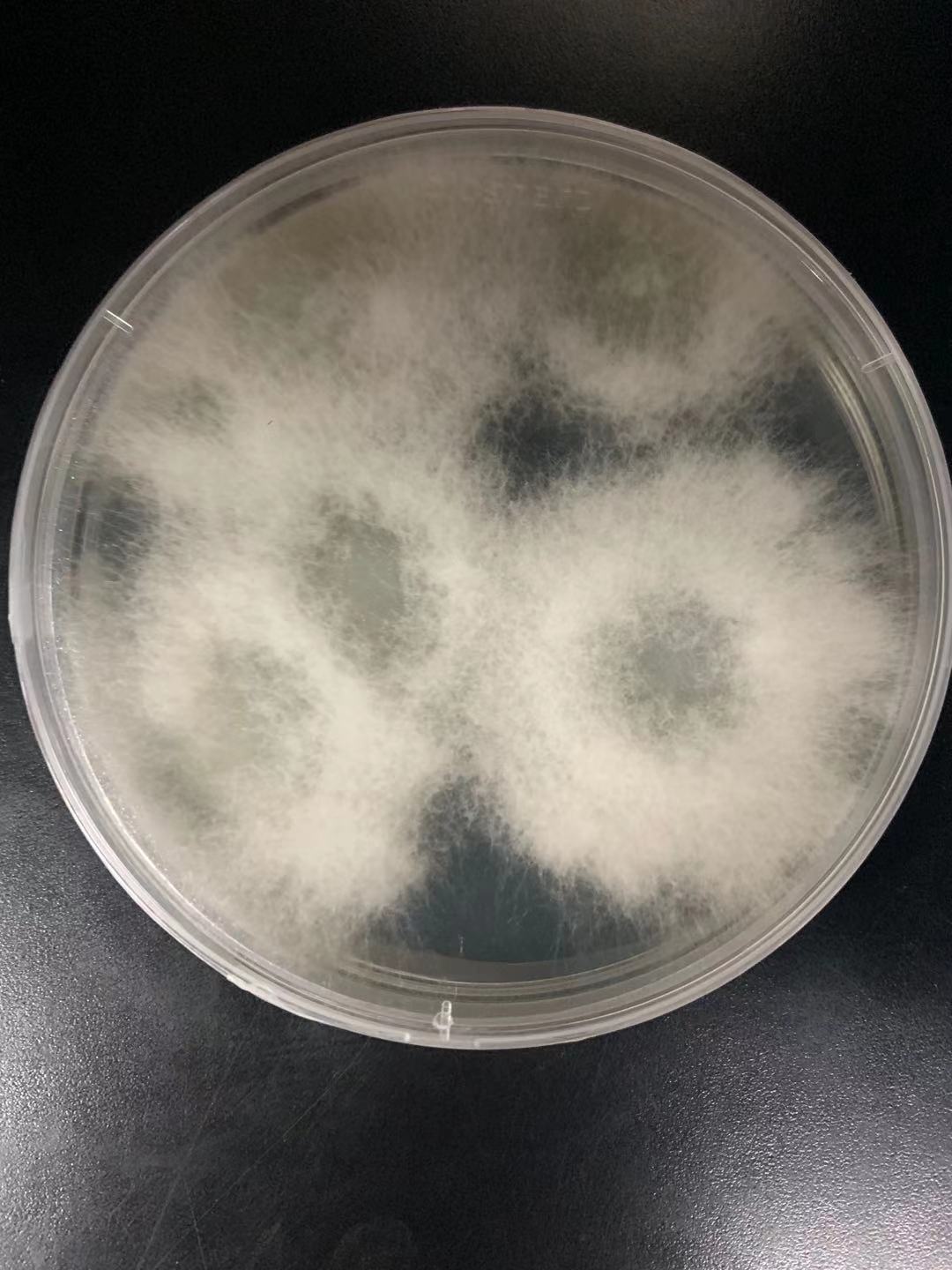

Supplement: Supplementary file 1 [file Data_Sheet_1.ZIP › Primary Data/Primary Data/Figure6/8RM.jpg]

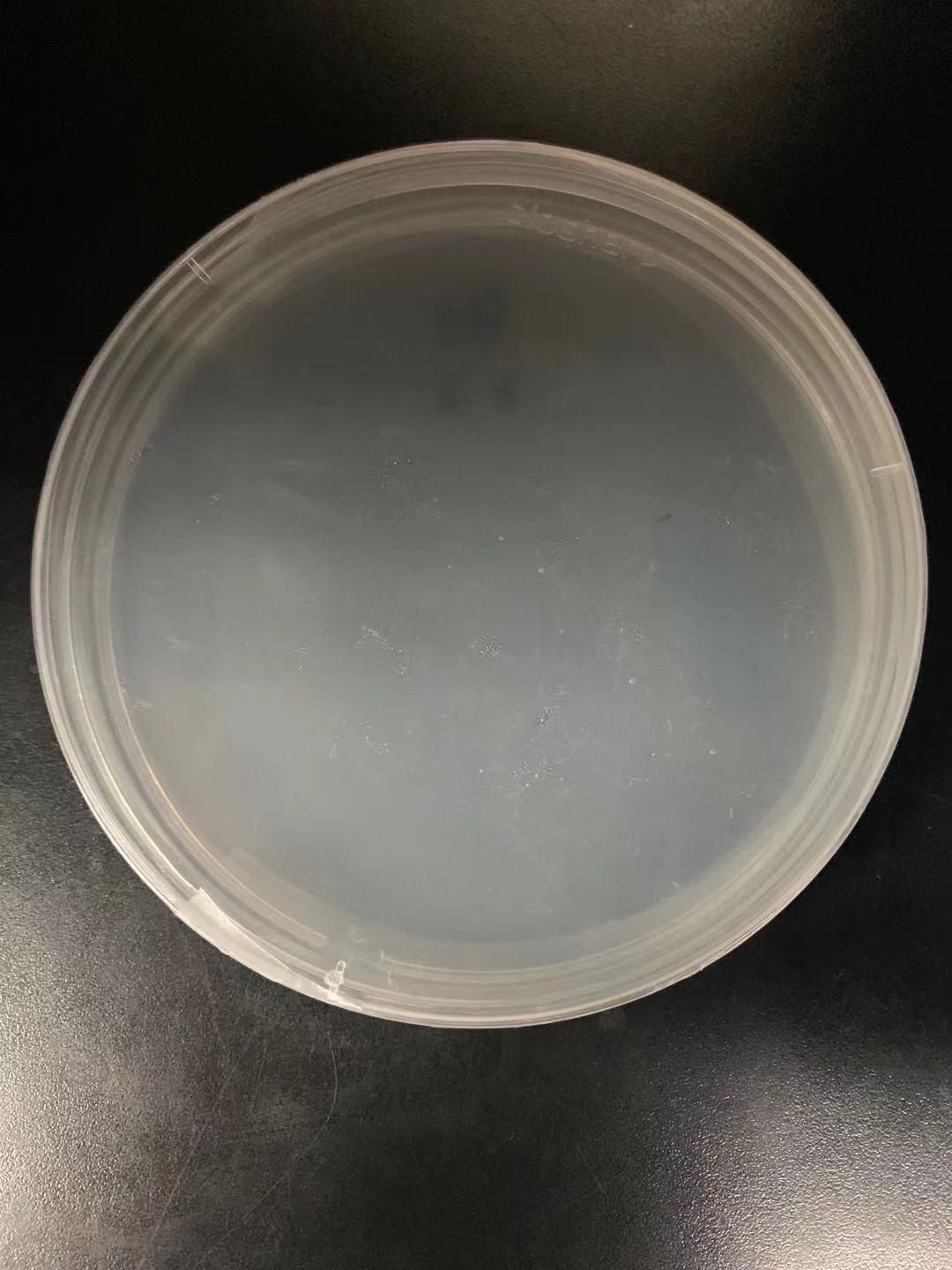

Supplement: Supplementary file 1 [file Data_Sheet_1.ZIP › Primary Data/Primary Data/Figure6/8SH.jpg]

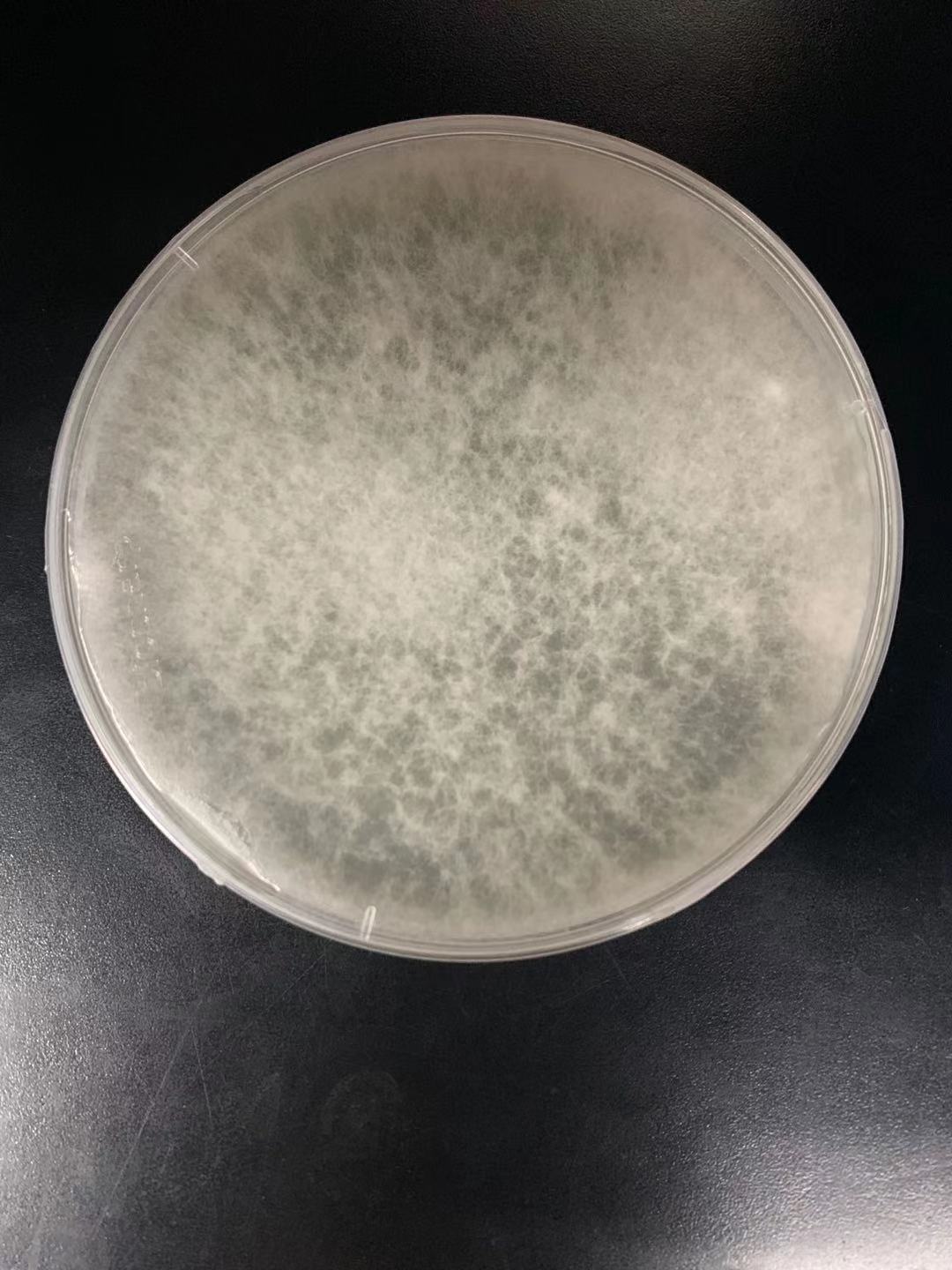

Supplement: Supplementary file 1 [file Data_Sheet_1.ZIP › Primary Data/Primary Data/Figure6/8SR.jpg]

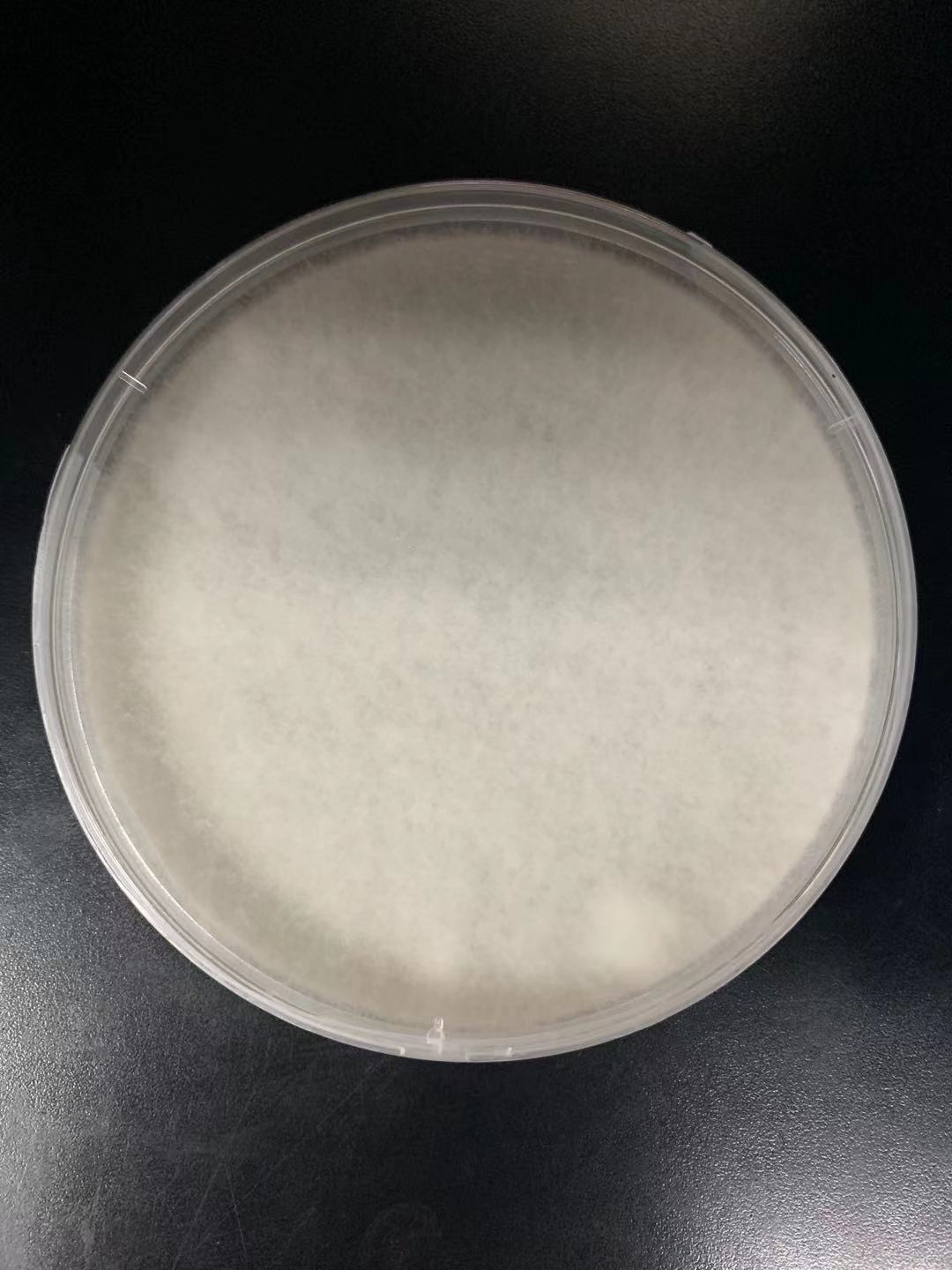

Supplement: Supplementary file 1 [file Data_Sheet_1.ZIP › Primary Data/Primary Data/Figure6/8YEPS.jpg]

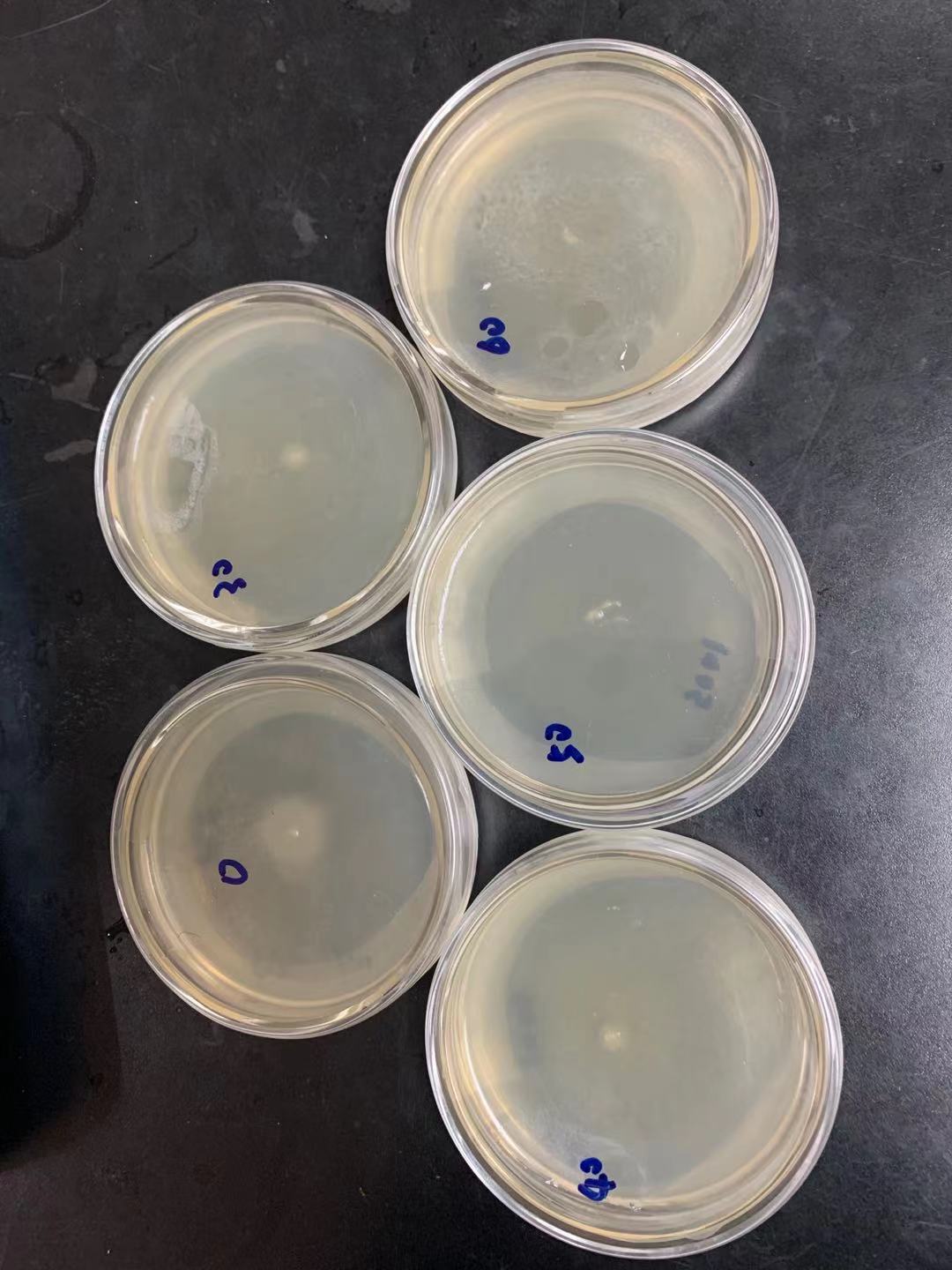

Supplement: Supplementary file 1 [file Data_Sheet_1.ZIP › Primary Data/Primary Data/Figure8/a.jpg]

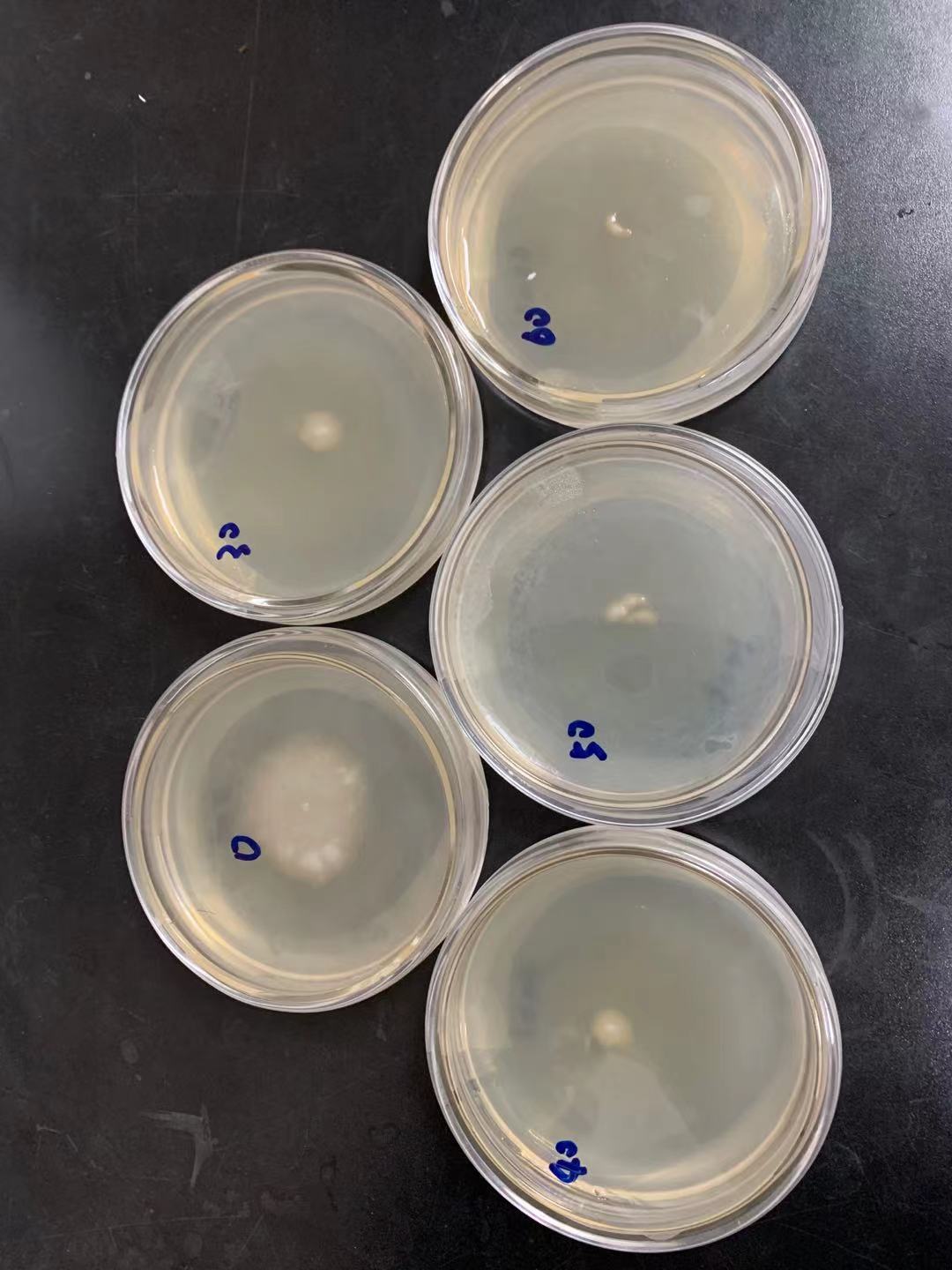

Supplement: Supplementary file 1 [file Data_Sheet_1.ZIP › Primary Data/Primary Data/Figure8/b.jpg]

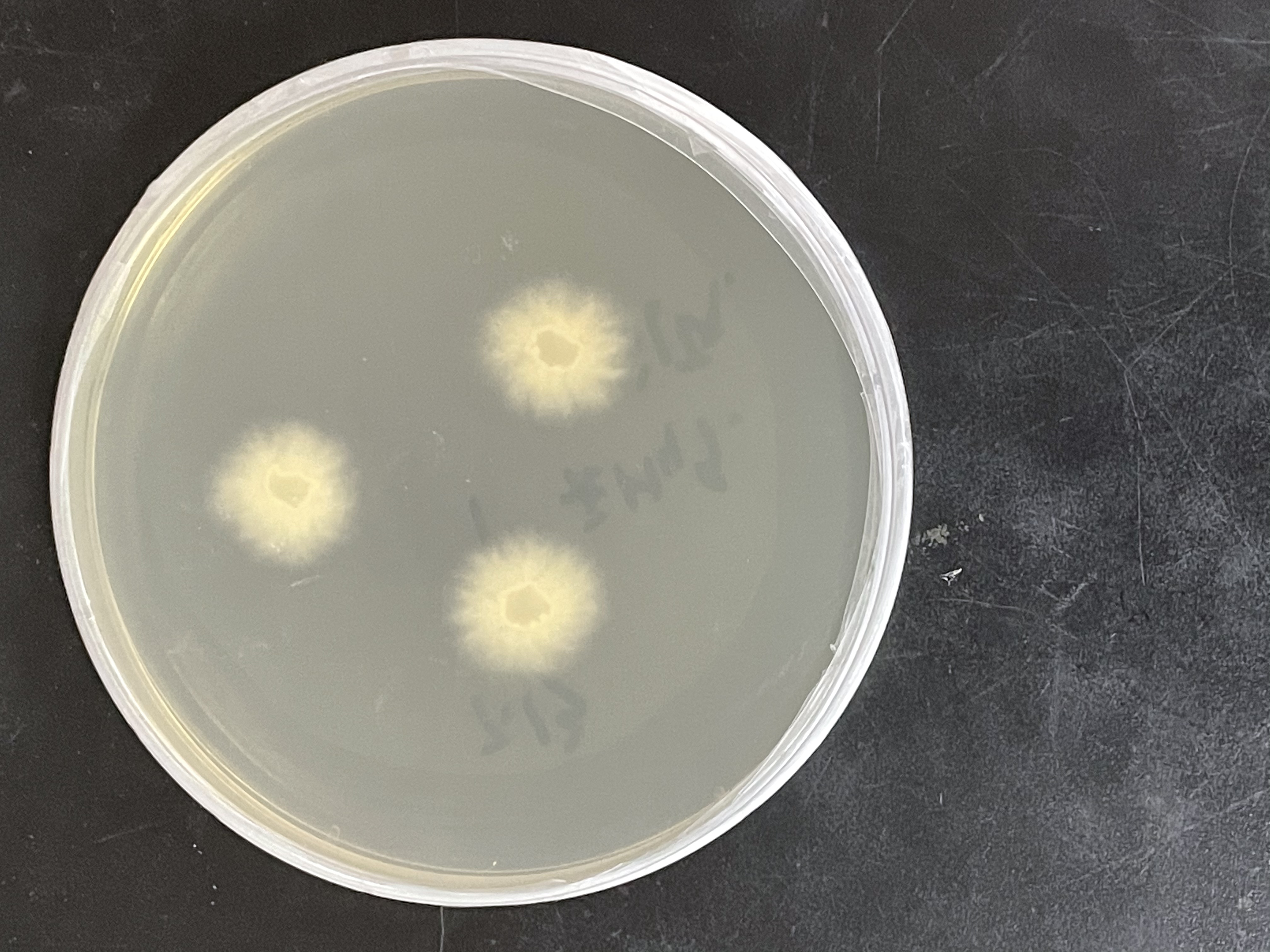

Supplement: Supplementary file 1 [file Data_Sheet_1.ZIP › Primary Data/Primary Data/Figure8/c.jpg]

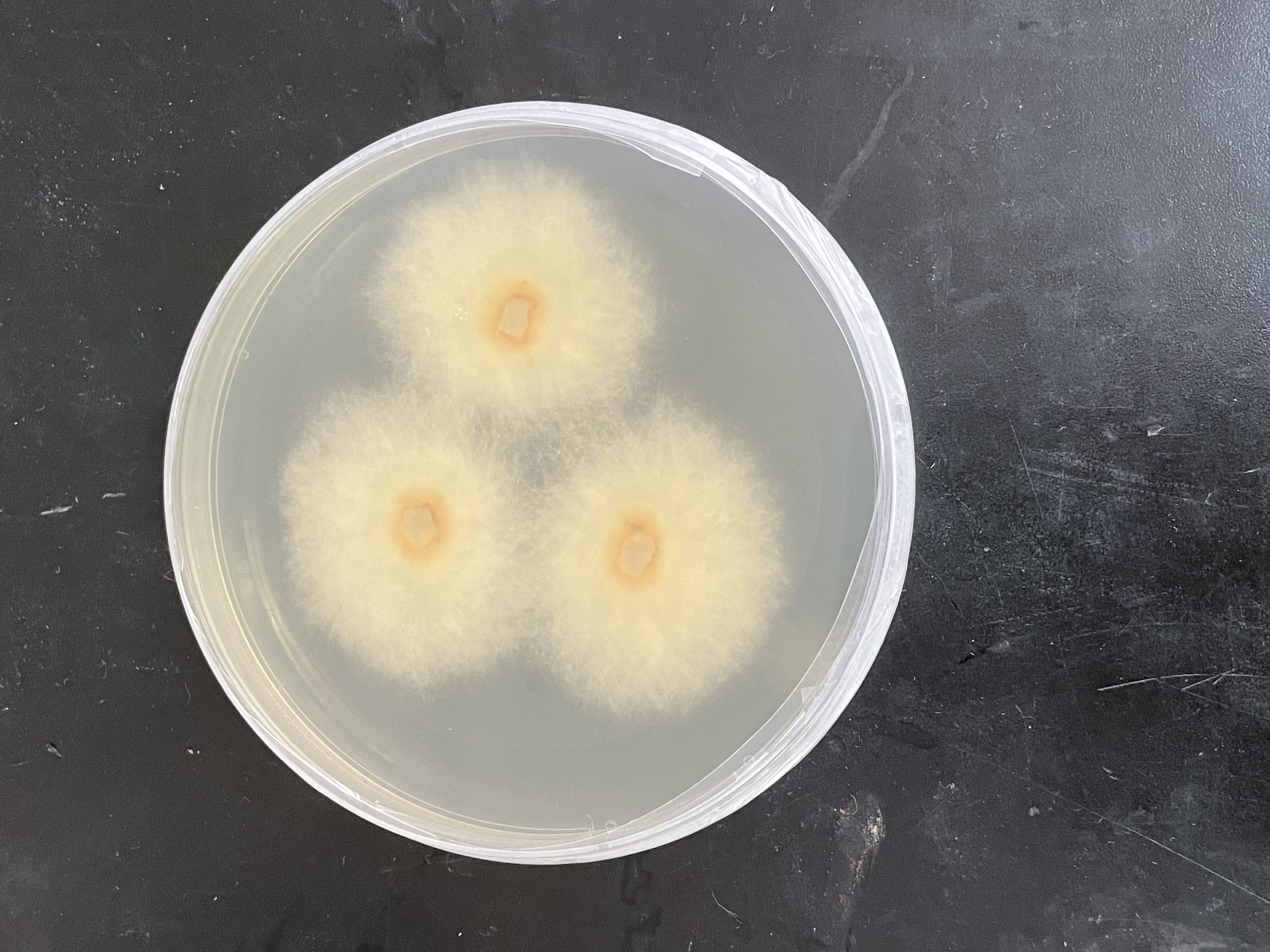

Supplement: Supplementary file 1 [file Data_Sheet_1.ZIP › Primary Data/Primary Data/Figure8/d.jpg]

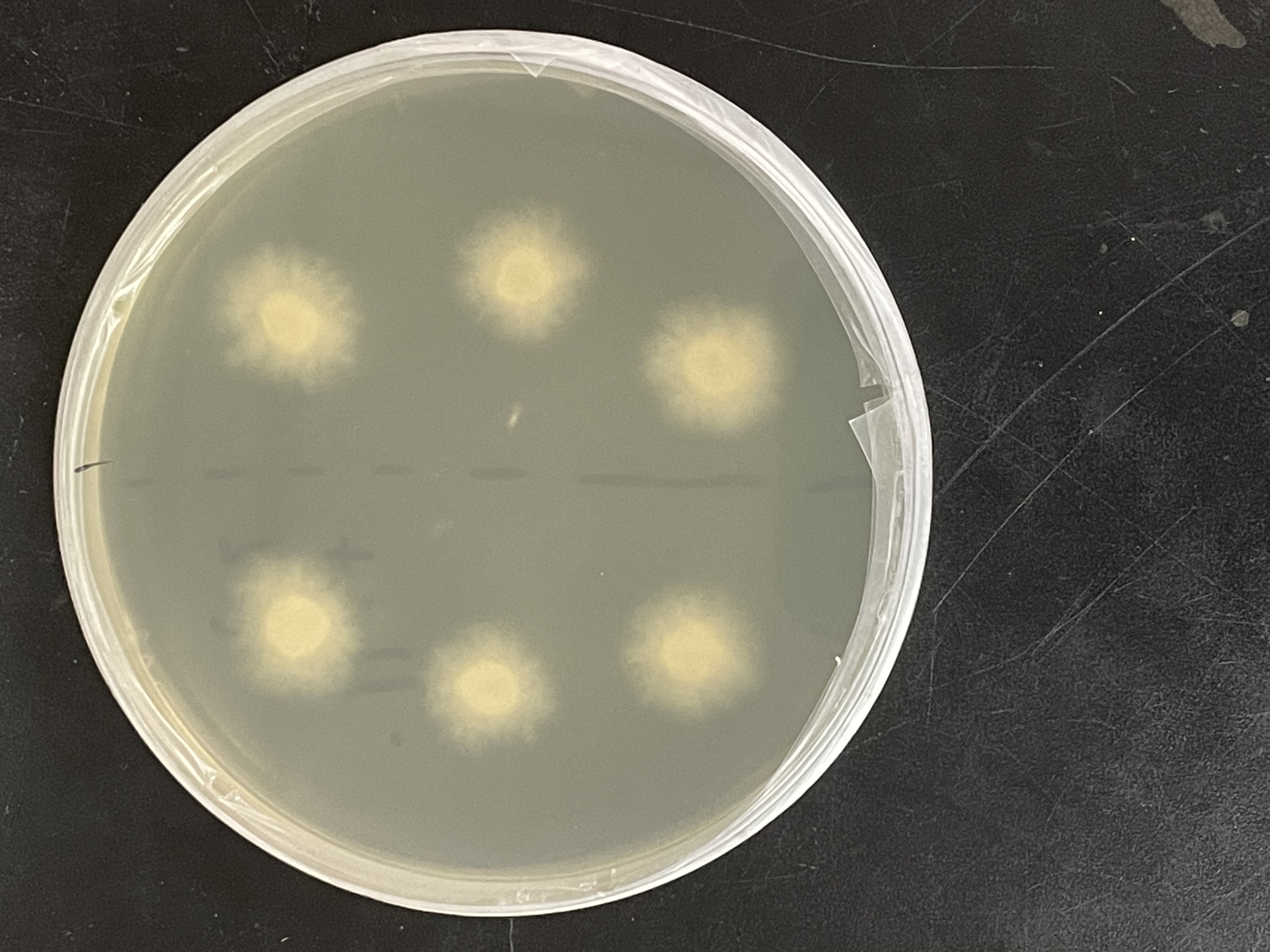

Supplement: Supplementary file 1 [file Data_Sheet_1.ZIP › Primary Data/Primary Data/Figure8/e.jpg]

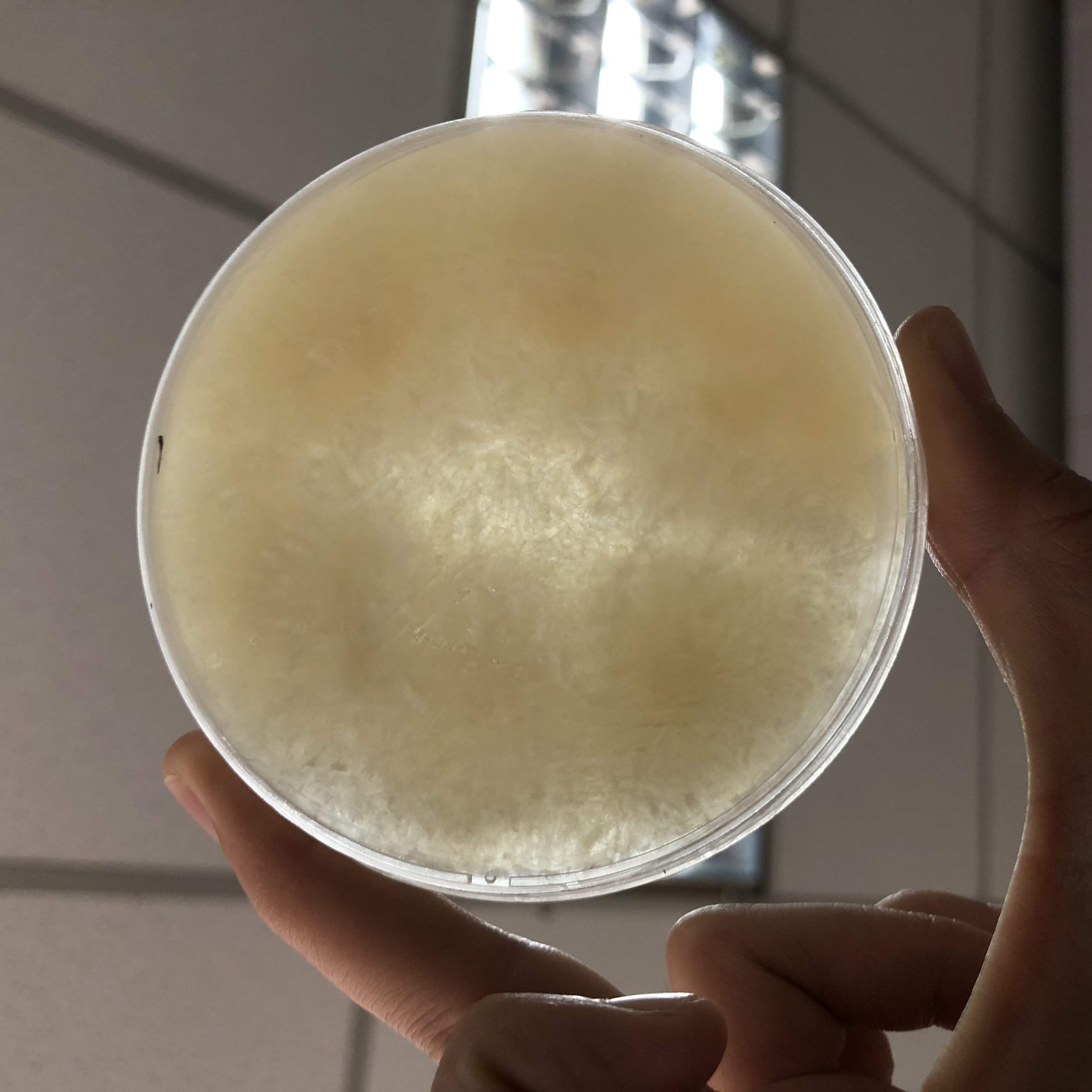

Supplement: Supplementary file 1 [file Data_Sheet_1.ZIP › Primary Data/Primary Data/Figure8/f.jpg]

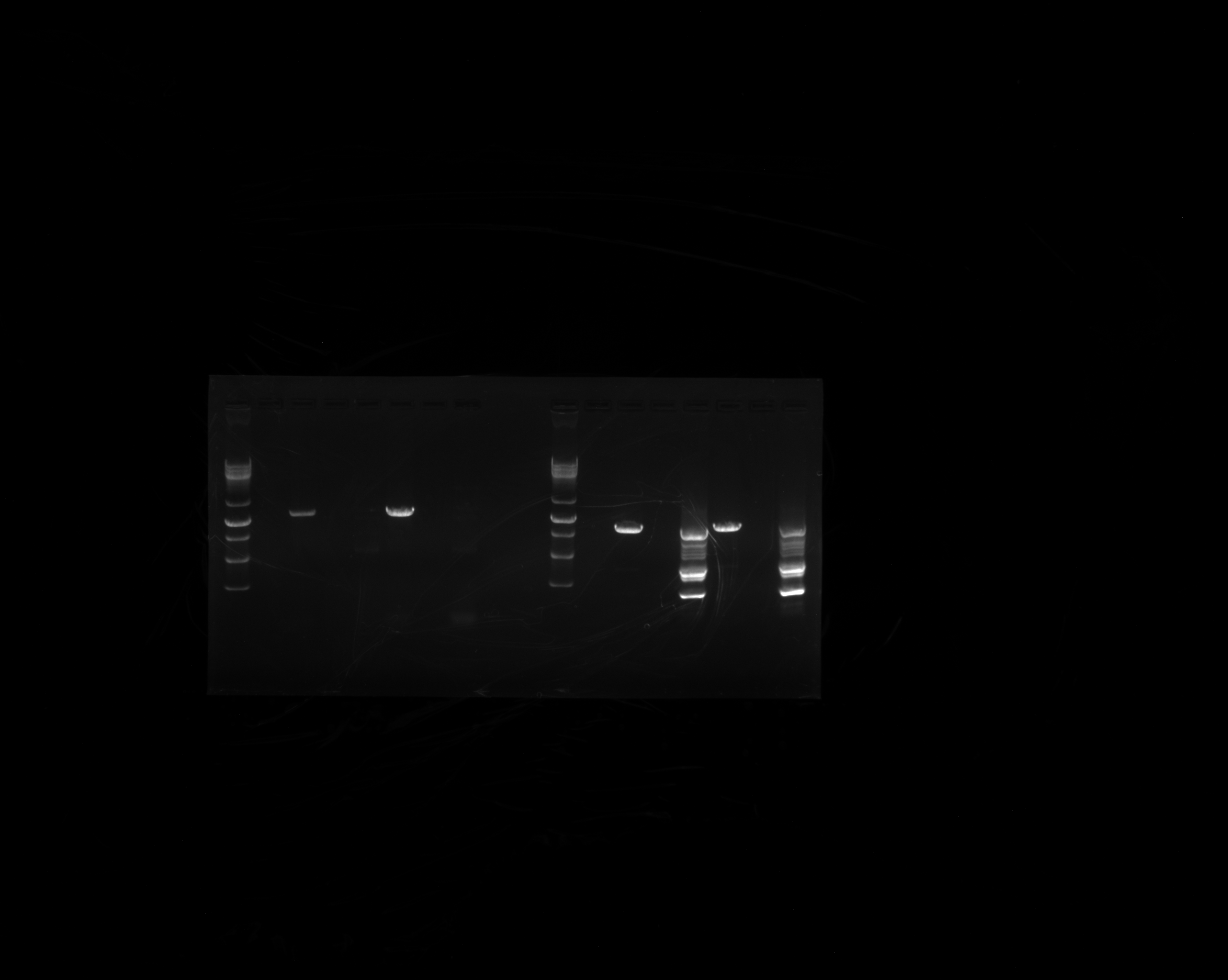

Supplement: Supplementary file 1 [file Data_Sheet_1.ZIP › Primary Data/Primary Data/Figure9/Figure9.tif]
