# Supplementary material for: Optimization of Protoplast Preparation and Establishment of Genetic Transformation System of an Arctic-Derived Fungus Eutypella sp
Source: Front Microbiol. 2022 Apr 6;13:769008. doi: 10.3389/fmicb.2022.769008 (PMC9019751; doi:10.3389/fmicb.2022.769008)
Supplement: Supplementary file 2 [file Data_Sheet_2.docx]

Supplementary Material

**Table S1**. PCR primer sets used in this study

| **Primers** | **Oligonucleotide Sequence (5’-3’)** |
| --- | --- |
| Ter-5-F(P1) | GTCAGATGCCCATCTTATCC |
| Ter-5-R(P2) | CCTTCAATATCATCTTCTGTCGAGATGACGATCTAGCAGGTAA |
| Ter-H-F(P3) | TTACCTGCTAGATCGTCATCTCGACAGAAGATGATATTGAAGG |
| Ter-H-R(P4) | GGCCGTTTCTGTTATACGCAAAGAAGGATTACCTCTAAACAA |
| Ter-3-F(P5) | TTGTTTAGAGGTAATCCTTCTTTGCGTATAACAGAAACGGCC |
| Ter-3-R(P6) | CCTAAGATGGCTACCAGCTA |
| H-F(P11) | TCGACAGAAGATGATATTGAAGG |
| H-R(P10) | AAGAAGGATTACCTCTAAACAA |

**Original Images for Gels**


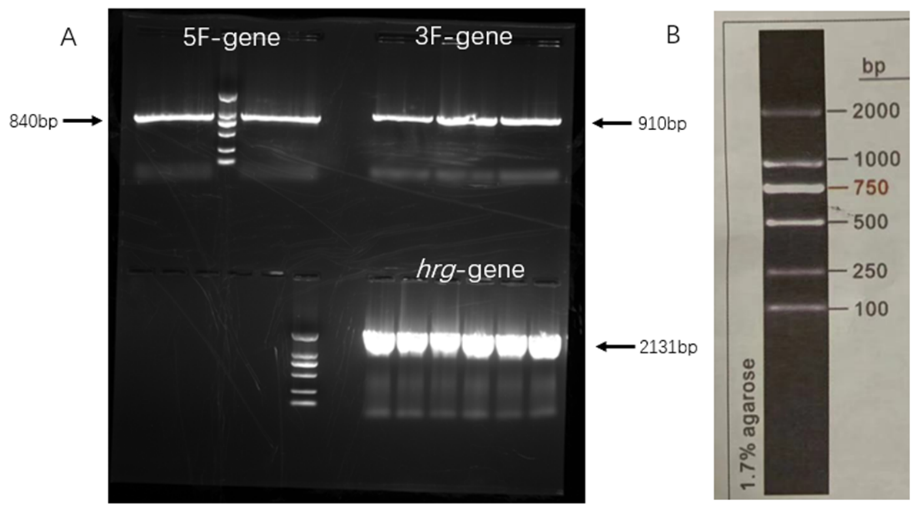


**Supplementary Figure S1**. Construction of deletion cassette by PCR. (**A**) Upstream and downstream of the target genes were amplified from genomic DNA of *Eutypella* sp. D-1 using designated primers, respectively (Table S1). The *hrg* marker fragment were amplified from pAg1-H3 using appropriate primers in Table S1. (**B**) Marker.


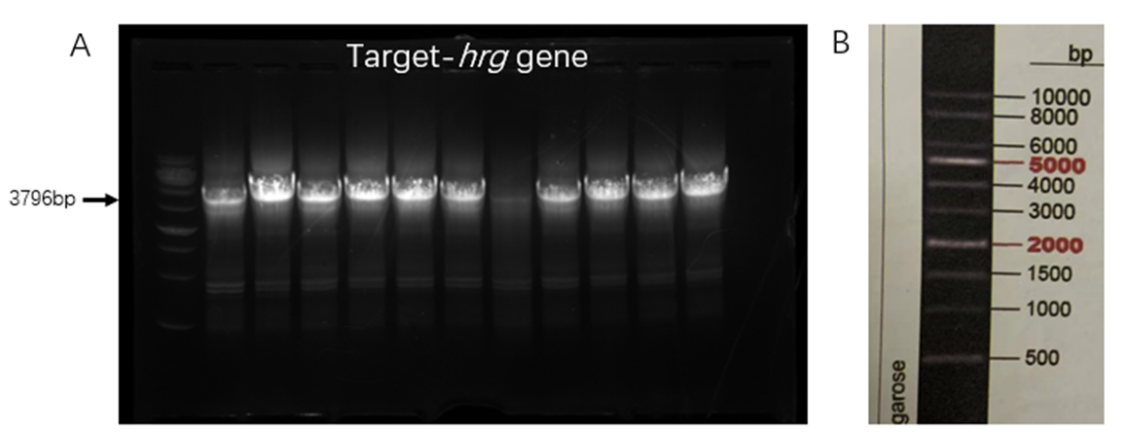


**Supplementary Figure S2.** Three purified PCR fragments (Figure S1) were assembled to yield the deletion cassette. (**A**) Deletion cassette (Target-*hrg* gene). (**B**) Marker.


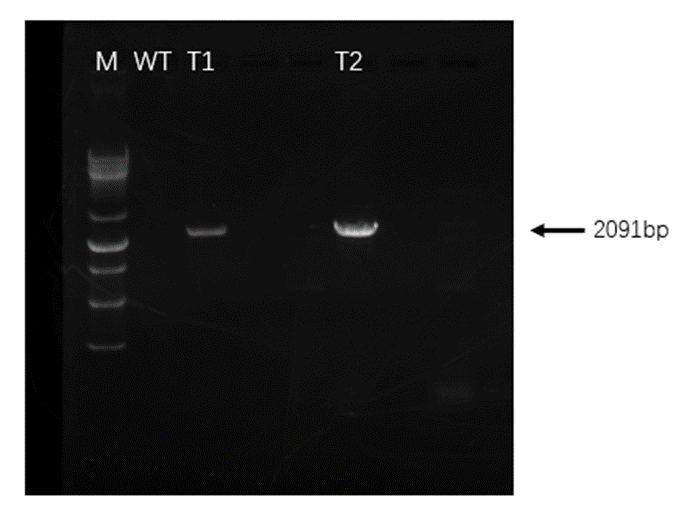


**Supplementary Figure S3**. Diagnostic PCR to identify the transformants. (M: marker; WT: wild-type strain; T1: transformant 1; T2: transformant 2).
